# Evolution of cooperation on temporal networks

**DOI:** 10.1038/s41467-020-16088-w

**Published:** 2020-05-08

**Authors:** Aming Li, Lei Zhou, Qi Su, Sean P. Cornelius, Yang-Yu Liu, Long Wang, Simon A. Levin

**Affiliations:** 1 Center for Systems and Control, College of Engineering, Peking University, Beijing, 100871 China; 2 Department of Zoology and Department of Biochemistry, University of Oxford, Oxford, OX1 3PS UK; 3 Center for Complex Network Research and Department of Physics, Northeastern University, Boston, MA 02115 USA; 4 Department of Ecology and Evolutionary Biology, Princeton University, Princeton, NJ 08544 USA; 5 Department of Mathematics and Department of Biology, University of Pennsylvania, Philadelphia, PA 19104 USA; 6 Department of Physics, Ryerson University, Toronto, ON M5B 2K3 Canada; 7 Channing Division of Network Medicine, Brigham and Women’s Hospital, Harvard Medical School, Boston, MA 02115 USA; 8 Center for Cancer Systems Biology, Dana-Farber Cancer Institute, Boston, MA 02115 USA

**Keywords:** Applied mathematics, Complex networks

## Abstract

Population structure is a key determinant in fostering cooperation among naturally self-interested individuals in microbial populations, social insect groups, and human societies. Traditional research has focused on static structures, and yet most real interactions are finite in duration and changing in time, forming a temporal network. This raises the question of whether cooperation can emerge and persist despite an intrinsically fragmented population structure. Here we develop a framework to study the evolution of cooperation on temporal networks. Surprisingly, we find that network temporality actually enhances the evolution of cooperation relative to comparable static networks, despite the fact that bursty interaction patterns generally impede cooperation. We resolve this tension by proposing a measure to quantify the amount of temporality in a network, revealing an intermediate level that maximally boosts cooperation. Our results open a new avenue for investigating the evolution of cooperation and other emergent behaviours in more realistic structured populations.

## Abstract (web summary)

Population structure enables emergence of cooperation among individuals, but the impact of the dynamic nature of real interaction networks is not understood. Here, the authors study the evolution of cooperation on temporal networks and find that temporality enhances the evolution of cooperation.

## Introduction

Explaining the evolution of durable, widespread cooperative behaviour in groups of self-interested individuals has been a challenge since the time of Darwin^1–8^. In response, researchers have turned to the critical role played by the underlying interaction networks, in which nodes represent individuals and links represent interactions^9,10^. It has been shown that the nontrivial population structures represented by both homogeneous^2–4,9,11–13^ and heterogeneous^10,14,15^ networks permit the formation of stable clusters of cooperators (altruists), which achieve higher individual payoffs while also resisting exploitation from defectors (egoists). As such, both theoretical analysis^4,9,10,15–20^ and behavioural experiments^21–28^ point to network structure as a key ingredient for the emergence of cooperation.

However, these and other deep insights about the evolution of cooperation generally rely on a key assumption that the underlying interaction network (contact graph) of individuals is time-invariant (i.e., static). In practice, this assumption is often violated, especially in social networks, which tend to be formed from an ever-changing amalgam of short-lived interactions. For example, emails and text messages represent near-instantaneous and hence ephemeral links in the corresponding temporal network^29^. Even in cases where interactions have non-negligible durations—such as phone calls, or the face-to-face interactions between inpatients in the same hospital ward—the network structure is in constant flux.

Recently, it has been shown that the temporality of edge activations can noticeably affect various dynamical processes, ranging from the information or epidemic spreading^30–33^ to network accessibility^34^ to controllability^35^. It is natural to expect that temporality will have a similarly profound effect in social systems, in which the relevant dynamical laws are strongly tied to the presence (or absence) of network links. There is a large body of work studying coevolutionary dynamics—in which the changes to the network structure are a direct result of the underlying dynamics (e.g., players strategically switching partners to shun defectors)^36–41^. Yet these mechanisms, though important, are just a few among many that influence the structure of real social networks, stressing the importance of studying temporality exogenous to the social dynamics. On this front, we mention one notable work that explored the impact of temporal social contacts on the evolution of cooperation, and claimed that the temporal dynamics of social ties favours selfish behaviour^42^. And yet, given some profound advantages of temporal networks recently discovered in the context of dynamics and control^35^, we are compelled to ask whether, under certain circumstances, temporality might actually enhance cooperation.

Here we study the evolution of cooperation on empirical and synthetic temporal networks first, and surprisingly, we find that temporal networks can facilitate the evolution of cooperation. We further investigate the impacts of bursty behaviour (namely, short timeframes of intense activity followed by long windows of relative silence)—a hallmark of many real social interaction patterns^43,44^. We find that this facet of temporality is actually detrimental to the emergence of cooperation, instead facilitating the spread of egoists. Finally, we rationalise the previous findings by introducing a measure of temporality in networks, and show analytically that an intermediate level most favours cooperation. We confirm the generality of our results over different types of synthetic networks, varying interaction time scales, updating rules (both synchronous and asynchronous), and game dynamics.

## Results

### Modelling framework

We conduct our investigation in the setting of classic evolutionary game theory^9,11,14^, in which two players interact by each choosing a strategy of cooperation (C) or defection (D). When their strategies agree, each player receives a payoff $R$ ($P$) for mutual cooperation (defection). When the players’ strategies disagree, the defector receives a payoff $T$ while the cooperator receives $S$. These outcomes can be encoded in the payoff matrix

$$\begin{array}{c} \quad {\mathrm{C}} \quad {\mathrm{D}}\\ \begin{array}{c}{\mathrm{C}}\\ {\mathrm{D}}\end{array}\left(\begin{array}{cc}R & S \\ T & P\end{array}\right)\end{array}$$

whose entries give the payoff under all possible combinations of strategies. For simplicity, we shall first focus on the widely-studied case of the (weak) Prisoner’s Dilemma^9,14,45,46^, which without loss of generality corresponds to the setting $R = 1,T = b$ and $S = P = 0$. This leaves a single temptation parameter, $b \, > \, 1$, which captures the potential advantage of defecting over cooperating^9^.

**Fig. 1:**
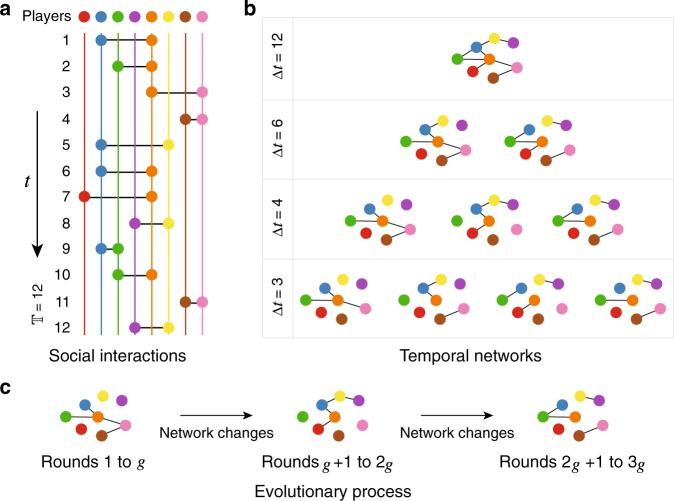
Construction of temporal networks from intermittent social interactions. **a** Social interactions between $8$ individuals indicated by solid circles with different colours. Along the whole time from $t = 1$ to $t = {\Bbb T}$, each individual is depicted by the same colour line, over which the corresponding circles will be given and connected with each other at time $t$ provided two players interact with each other during the time interval $(t - \tau ,t]$. Here $\tau = 1$ for the simplicity of visualisations, and normally in the real data collected by SocioPatterns (see Methods), $\tau = 20$s. **b** Four different temporal networks that arise from aggregating the interactions shown in **a** into snapshots using different time windows $\Delta t$. When $\Delta t = {\Bbb T}$, all interactions are captured in a single snapshot, corresponding to the static network that is the typical object of study in social network data. In general, when $\Delta t \, < \, {\Bbb T}$, we have $\left\lceil {{\Bbb T}/\Delta t} \right\rceil$ snapshots. **c** The definition of evolutionary process on temporal networks. Taking the temporal network corresponding to $\Delta t = 4$ in (**b**) as an example, we perform $g$ rounds of evolution in each snapshot before changing the network structure to the next one, and totally we run $G$ rounds. If $\left\lceil {{\Bbb T}/\Delta t} \right\rceil g \, < \, G$, we repeat the sequence of snapshots from the beginning.

Figure 1 illustrates the essence of our modelling framework. We consider the above game played out between pairs of adjacent nodes on a time-varying network, which we represent by a sequence of separate networks (snapshots) on the same set of $N$ nodes. Starting from empirical contact sequences (i.e. timestamped interactions), these snapshots are constructed by aggregating social contacts over successive, non-overlapping windows of $\Delta t$ (Fig. 1a and b), which determines the set of links active in a given snapshot. As a point of comparison, we also create a corresponding static network by aggregating all social contacts in the dataset.

To capture the interactions occurring on these networks, we initially set an equal probability for each individual (node) to choose C or D in the population on the first snapshot. In each round, every individual $i$ plays the above game with each of its neighbours, accumulating a total payoff $P_i$. Afterwards, the player may change his or her strategy by randomly imitating that of a neighbour. In our simulations, we employ a commonly used updating rule that models a tendency to imitate success^14^. Specifically, each player $i$ may pick a neighbour $j$ (having payoff $P_j$) from its $k_i$ neighbours, and then imitate $j$’s strategy with probability $(P_j - P_i)/(Dk_d)$ provided $P_j \, > \, P_i$. Otherwise, player $i$ keeps his/her current strategy. Here $D = T - S$ and $k_d$ is the larger of $k_i$ and $k_j$. We repeat this procedure a total of $g$ times before changing the network structure to the next snapshot (Fig. 1c). In this way, $g$ is a parameter that controls the timescale difference between the dynamics on the network versus the dynamics of the network. We continue running the game for a total of $G$ rounds, and then measure the average fraction of cooperators ($f_{\mathrm{c}}$) over another 2,000 rounds, similar to the canonical procedure used in static networks^14,15^. Note that, in a departure from previous studies^36–41^, here the time-varying nature of the networks is completely exogenous, not being coupled to the game dynamics (by, for example, players changing whom they interact with to shun defectors). This allows us to independently study the effect of network temporality on the dynamics of the game.

### Temporal networks facilitate the evolution of cooperation

**Fig. 2:**
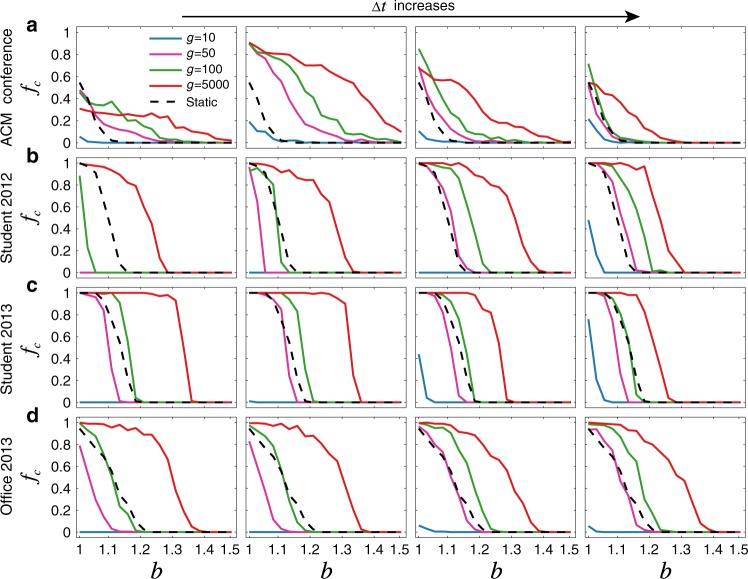
Temporal networks generally promote the evolution of cooperation in real social systems. For four empirical datasets: **a** the ACM conference, **b** Student 2012, **c** Student 2013, and **d** Office 2013, we show the frequency of cooperation ($f_{\mathrm{c}}$) on both temporal (coloured lines) and static (black dashed lines) networks with different values of the aggregation time windows $\Delta t$. We choose $1,2,6,24$ h from left to right in **a** to **c** and $6,8,12,24$ h in **d**, respectively. After letting the population evolve $g$ rounds on each snapshot, we average over another $2000$ rounds after a transient time of $G = 10^6$ rounds on each temporal network, to obtain the equilibrium frequency of cooperators. The statistics of each dataset are given in Supplementary Table 1.

Our principal result is that temporal networks generally enhance cooperation relative to their static counterparts. What’s more, they allow it to persist at higher levels of temptation, $b$. Figure 2 shows the equilibrium fraction of cooperators $f_{\mathrm{c}}$ for temporal networks formed from social contacts in four empirical datasets: attendees at a scientific conference (ACM conference)^47^, students at a high school in Marseilles, France in two different years^48,49^ (Student 2012, and Student 2013), and workers in an office building in France (Office 2013)^50^. In each of these systems we observe a broad range of $g$ over which $f_{\mathrm{c}}$ is greater in the temporal network than in its static counterpart, at almost all values of $b$. Strikingly, this is true even for small $\Delta t$; in this case the network’s links are distributed over a large number of snapshots, leaving little network scaffolding on which to build stable clusters of cooperators. Nonetheless, there exists a range of $g$ that can compensate for this sparsity, again giving temporal networks the victory in terms of enhancing cooperation. Indeed, we find that the only scenario in which temporal networks result in less cooperation than static networks is when $g$ is small. In this limit, the evolutionary timescale is comparable to the dynamical timescale, and patterns of cooperation have no time to stabilise before being disrupted by the next change in network structure. This squares our results with the previously-mentioned conclusion that temporality inhibits cooperation (Supplementary Fig. 1), which was obtained from the regime of a single game per snapshot ($g = 1$) with comparatively infrequent strategy updates^42^. Interestingly, regardless of the value of $g$, our simulations show a rapid and on-average monotonic convergence of the cooperator fraction toward equilibrium (Supplementary Fig. 2)—similar to the temporal profile previously found in co-evolving random networks^51^.

As there are many factors that might affect evolutionary outcomes^15,52,53^, we have studied numerous alternative setups as well. These include: (i) using the original time scale of network edges (Supplementary Fig. 1), (ii) asynchronous updating of strategies (Supplementary Fig. 3), (iii) alternative social dilemmas like the canonical Stag-Hunt ($S \, < \, P \, < \, T \, < \, R$) and Snowdrift ($P \, < \, S \, < \, R \, < \, T$) games, and the general Prisoner’s Dilemma with $S \, < \, P$ (Supplementary Figs. 4 and 5), and finally (iv) a different strategy update rule that allows players to imitate worse-performing neighbours^54^ (Supplementary Fig. 6). None of these modifications alter our main finding that time-varying network structure generally enhances the evolution of cooperation.

**Fig. 3:**
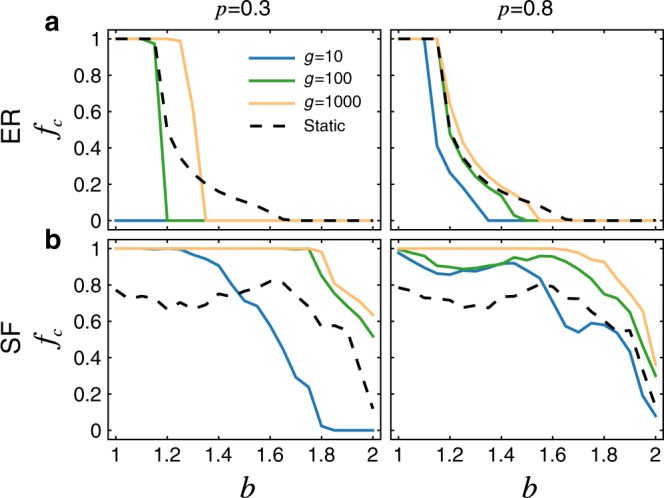
Evolution of cooperation on temporal networks generated from synthetic data. Here we generate $M$ sparse snapshots based on: **a** a base Erdős-Rényi (ER) random network^55^; and **b** a base scale-free (SF) network with degree exponent $2.5$ constructed by the static model^70^, choosing a fraction $p$ of edges to be active within each snapshot. Note that when $p$ is bigger, there are more links being active in each snapshot, which reduces the gap of the results obtained from temporal and static networks, while large $g$ does not necessarily reduce the gap (Supplementary Fig. 9). Here $M = 100$, the network size $N = 1000$, and average degree $\langle k\rangle = 10$. The robustness of the corresponding results for other parameters and other methods of generating synthetic temporal networks has been verified (see Supplementary Fig. 7).

To test whether this result depends on idiosyncrasies of the temporal patterns in real social systems, we have also simulated games on synthetic temporal versions of Erdős-Rényi (ER)^55^ and scale-free (SF)^56^ networks (see Methods). Here too we find that with almost any level of temporality, cooperators have an easier time gaining footholds in the population (Fig. 3). Interestingly, we find that temporal versions of SF networks yield a higher $f_{\mathrm{c}}$, all other things being equal, than the temporal ER networks (Fig. 3 and Supplementary Fig. 7). As such, the well-known result that heterogeneous degree distributions enhance cooperation in static networks also holds in temporal networks^14^. Note that our results here are robust to changes in the size and average connectivity of the networks under consideration (Supplementary Figs. 7 and 8).

### Effects of burstiness on the evolution of cooperation

Analyses of the temporal patterns of human interactions in email^57^, phone calls^57,58^, and written correspondence^43^ have revealed a high degree of burstiness—periods of intense activity punctuated by relative lulls—resulting in a heavy-tailed inter-event time distribution^43^. Such temporal correlations in activity have been shown to have effects on network dynamics above and beyond those of temporality alone, for instance accelerating the spread of contagions^59,60^. We have established that burstiness is present to varying degrees in each of the four datasets we study (Supplementary Fig. 10), prompting us to ask whether it helps or hinders the evolution of cooperation.

**Fig. 4:**
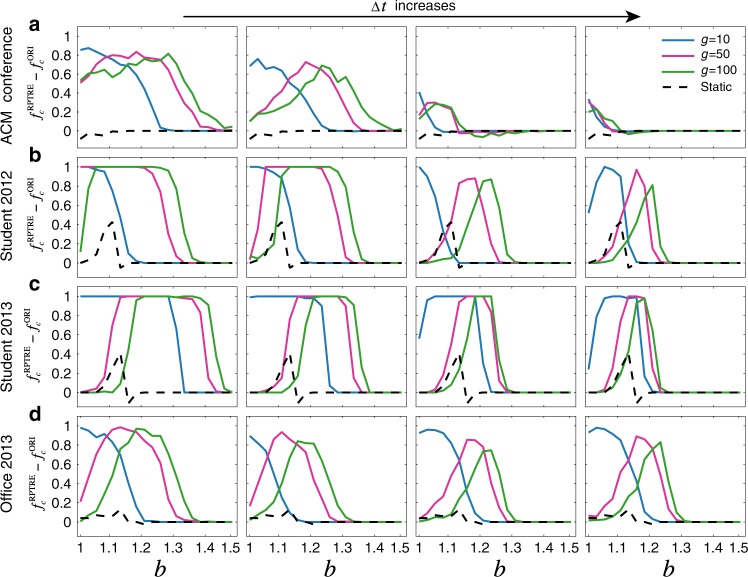
The intrinsic bursty behaviour in human interactions suppresses the maintenance of cooperation. For each dataset, we show the difference $f_{\mathrm{c}}^{{\mathrm{RPTRE}}} - f_{\mathrm{c}}^{{\mathrm{ORI}}}$ between the frequency of cooperators $f_{\mathrm{c}}^{{\mathrm{RPTRE}}}$ in temporal networks generated from each datsaset after randomly permuting both the timestamps and edges (RPTRE) which erases the burstiness inherent to human interaction data (see Methods), and $f_{\mathrm{c}}^{{\mathrm{ORI}}}$ over the original scenarios. By construction, at any fixed value of $b$, each curve here sums to at most $1$ with the corresponding curve in Fig. 2 from **a**–**d**. We see that the frequency of cooperators generally increases after the bursty behaviour is destroyed, suggesting that correlations in activity within a social network are antagonistic toward the formation of cooperation. Note that for clarity of presentation, we did not plot the case for $g = 5000$. However, all results for each dataset after randomisations with different null models^29^ can be found in Supplementary Figs. 11–14. Other parameters are the same as those in Fig. 2.

We address this question by shuffling each dataset, randomising the source, target, and timestamp of each social contact (see Methods). We stress that this randomisation has the effect of erasing bursty behaviour at the level of individual nodes. Figure 4 shows that, in every temporal network we consider, cooperation is improved after randomisation, suggesting that bursty behaviour impedes the evolution of cooperation. Indeed, due to the heterogeneity of active times embedded in bursty behaviour of different players, it is harder for cooperators to form stable clusters to obtain benefits from mutual cooperation in order to compensate for the losses against defectors^9,25^. For the effects of other null models that permute only the structure or the time stamps of the contacts, please refer to Supplementary Figs. 11–14, where we also show that the above results are robust to alternative randomisation protocols. Furthermore, this is true for nearly all choices of parameters $\Delta t$, $g$, and $b$. But how do we reconcile the fact that burstiness is inimical to cooperation with our previous observation, namely that temporality generically promotes it?

### Cooperation is maximised at intermediate temporality

The burstiness and the parameters $g$ and $\Delta t$ encode three different facets of temporality. Specifically, $g$ captures the relationship between the dynamical/structural timescales; $\Delta t$ on the other hand indicates the extent to which the network structure is spread over time; finally, the burstiness represents time correlations in the network structure. To understand the effects of these parameters in a unified way, we define the following measure of the temporality ${\cal{T}}$ of a temporal network with $M$ snapshots as

$${\cal{T}} = \frac{1}{{M - 1}}\mathop {\sum}\limits_{m = 1}^{M - 1} {\frac{{\mathop {\sum}\nolimits_{i,j} {| {a_{ij}(m) - a_{ij}(m + 1)} |} }}{{\mathop {\sum}\nolimits_{i,j} {{\mathrm{max}}\{ {a_{ij}(m),a_{ij}(m + 1)} \}} }}} .$$

Here $a_{ij}(m)$ is the connectivity between nodes $i$ and $j$ in snapshot $m$, being $1$ if the nodes have a contact in the associated time window and $0$ otherwise; the above fraction equals $0$ for any two nearby empty networks without links. This measure captures the tendency of a randomly-chosen link to change status (either active to inactive, or vice versa) in the next snapshot. By construction, we always have $0 \, < \, {\cal{T}} \le 1$, with ${\cal{T}} = 0$ occurring in the limit where network topology does not change (i.e. a static network), and ${\cal{T}} = 1$ corresponding to the case where all links in a given snapshot are different from those in the previous snapshot.

**Fig. 5:**
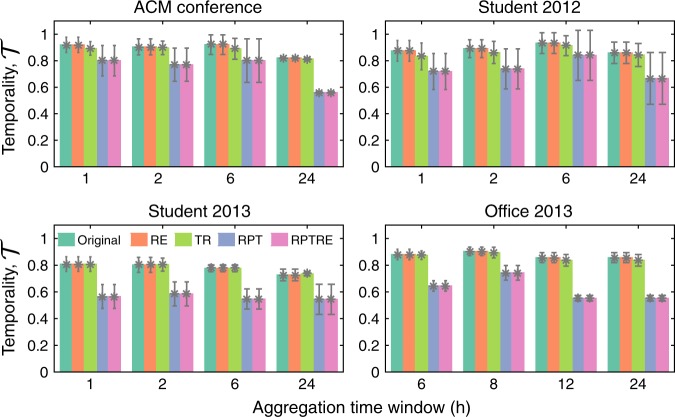
The temporality of real networks. The temporality ${\cal{T}}$ of the original datasets is shown alongside their randomisations for different time windows $\Delta t$. By destroying burstiness, randomisations altering the time ordering of contacts (RPT, RPTRE) decrease ${\cal{T}}$. Supplementary Fig. 15 shows how the overall temporality ${\cal{T}}$ arises on a snapshot-by-snapshot basis, and the corresponding standard deviation is given in this figure as the error bar.

Figure 5 shows the values of ${\cal{T}}$ for both the original and randomised versions of each of the four datasets we study. We see that at $\Delta t = 1$, the original data tend to display high temporality, which decreases upon randomisation, suggesting that most interactions (links) in these systems last less than $1$ hour. Considering our earlier finding that the cooperation level $f_{\mathrm{c}}$ increases after randomisation (Fig. 4), this suggests that too-high temporality hinders the spread of cooperation, instead fostering egoistic behaviour. On the other hand, we find that a too-low value for ${\cal{T}}$ is also associated with diminished cooperation (Supplementary Fig. 16). Altogether, the picture that emerges is one of an intermediate regime—a sweet spot of temporality at which cooperation is maximally enhanced relative to static systems.

### Theoretical analysis

Having demonstrated that an intermediate level of $\Delta t$ facilitates cooperation most, we are prompted to theoretically explain this observation. We model temporal networks theoretically using the activity-driven model^33^. Here, a node can either be active—in which case it forms links with an average $l$ randomly-chosen other nodes—or inactive, in which case its links (if any) come from other active nodes. We denote by $a_i$ the probability that node $i$ is active in a given snapshot. Let $N_a$ denote the total number of players in the snapshot $m$ having a specified activity value $a$, and let $D_a^m$ denote the expected number of those that are defectors. Also, we will denote by $\mu$ ($\lambda$) the average probability for a defector (cooperator) to become a cooperator (defector) in the next round.

Staring from a specific snapshot $m$, the number of defectors in the next snapshot, $D_a^{m + 1}$ arises as a sum of three contributions: (a) defectors in the current snapshot (i.e. $D_a^m$), minus (b) the number of them that convert to cooperators (i.e. $\mu D_a^m$), plus (c) the new defectors converted from cooperators in the last round. Note that, in (c), new defectors can arise either as (i) active cooperators who interact with (and imitate) neighbouring defectors (i.e. $(N_a - D_a^m)al\left( {{\int} {\mathrm{d}}a^{\prime}D_{a^{\prime}}^m/N} \right)\lambda$), or (ii) inactive cooperators who nonetheless share a link with an active defector and imitate that defector’s strategy (i.e. ${\int} {{\mathrm{d}}a^\prime D_{a{^\prime}}^ma^\prime l\left[ {\left( {N_a - D_a^m} \right)/N} \right]\lambda }$, where $(N_a - D_a^m)/N$ is the probability that cooperators with active probability $a$ are selected to interact with an active defector). Combining these contributions, we can write a self-consistent equation for the evolution of $D_a^m$

1 $$D_a^{m + 1} = D_a^m - \mu D_a^m + \left( {N_a - D_a^m} \right)al{\int} {{\mathrm{d}}a^\prime D_{a^{\prime}}^m\frac{\lambda }{N}} + {\int} {{\mathrm{d}}a^\prime D_{a^{\prime}}^m a^{\prime} l\frac{{N_a - D_a^m}}{N}\lambda } .$$

When we take the continuum limit with respect to the time $m$, the above equation corresponds to the following system of differential equations

2 $$\left\{ {\begin{array}{*{20}{l}} {\partial _mD = - \mu D + \lambda l\langle a\rangle D + \lambda lQ} \hfill \\ {\partial _mQ = - \mu Q + \lambda l\langle a^2\rangle D + \lambda l\langle a\rangle Q} \hfill \end{array}\, ,} \right.$$

where $D$ is the number of defectors, $Q = {\int} {\mathrm{d}}aD_aa$, and $\langle a\rangle$ ($\langle a^2\rangle$) is the first (second) moment of $a$ over all players. The first expression in Eq. (2) is obtained by integrating over all values of $a$ and ignoring the second order terms (i.e. $Q^mD^m$ here). The second expression comes from multiplying both sides of Eq. (2) by $a$ and then integrating out (see Supplementary Note 1 for details). Regardless of the parameter values, this system possesses an equilibrium at $D = Q = 0$, corresponding to the complete absence of defectors. We can determine its stability by linearising to obtain the corresponding Jacobian matrix

$$J = \left( {\begin{array}{*{20}{l}} { - \mu + \lambda l\langle a\rangle } \hfill & {\lambda l} \hfill \\ {\lambda l\langle a^2\rangle } \hfill & { - \mu + \lambda l\langle a\rangle } \hfill \end{array}} \right),$$

which has eigenvalues $- \mu + \lambda l\langle a\rangle \pm \lambda l\sqrt {\langle a^2\rangle }$. When the largest eigenvalue is positive (equivalent to $\lambda /\mu \, > \, 1/[(\langle a\rangle + \sqrt {\langle a^2\rangle } )l]$), the equilibrium is unstable, meaning that defectors can never die out in the population. Interestingly, this equation is consistent with the epidemic threshold previously derived for activity-driven temporal networks^33^, where there $\lambda$ is the infection rate per contact and $\mu$ is the recovery rate. Indeed, whether defection or infection, the denominator captures the rate of spread of a small perturbation around an equilibrium (in this case $D = 0$), with both density ($\langle a\rangle$) and degree heterogeneity ($\langle a^2\rangle$) facilitating that spread.

The threshold for defection to gain a foothold in the population can be related to the network structure as follows. Considering that the average number of links for each player is $k = 2l\langle a\rangle$, we know that the probability for a defector to spread its strategy is $\lambda k$. Hence a nonzero fraction of defectors will break out if $\lambda k/\mu \ge {\cal{D}}^ \ast$, where ${\cal{D}}^ \ast$ is the threshold defined by

3 $${\cal{D}}^ \ast = \frac{{2\langle a\rangle }}{{\langle a\rangle + \sqrt {\langle a^2\rangle } }}.$$

We see that the increase of defectors is triggered (inhibited) by $\lambda$ ($\mu$), where the bigger (smaller) $\lambda$ ($\mu$) is, the more cooperators (defectors) switch to be defectors (cooperators). Therefore, beyond the criterion $\lambda k/\mu \ge {\cal{D}}^ \ast$ governing the existence of defectors, ${\cal{D}}^ \ast$ quantifies the difficulty for defectors to take over the whole network. Numerical validations for this threshold are shown in Supplementary Fig. 17, where we also show our analytical approximations of this threshold agree with the findings of canonical evolutionary dynamics in the case where a strategy’s payoff determines the change of its frequency.

**Fig. 6:**
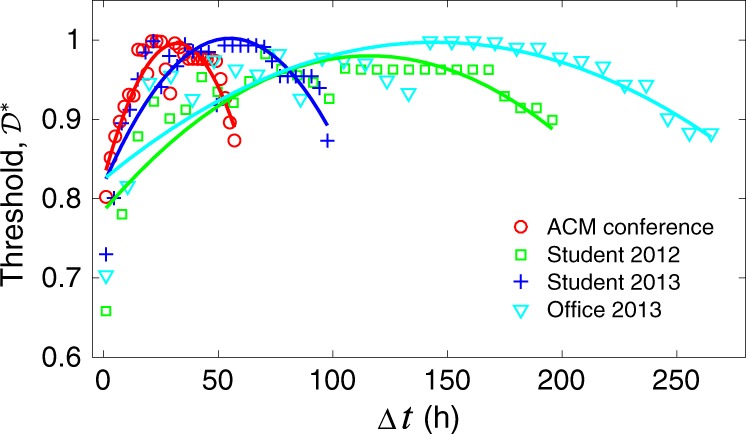
The threshold for the outbreak of defection on temporal networks reaches its maximum at the intermediate aggregation time windows. For all empirical datasets we considered, the corresponding thresholds governed by Equation (3) are presented numerically in scatter plots over the different aggregation time windows $\Delta t$. The corresponding lines are given by least square quadratic regression with $R^2 \, > \, 0.7$. Note that, for each dataset, the maximum $\Delta t$ is the total time the corresponding dataset covers (see Supplementary Table 1).

Equation (3) tells us that the defection threshold in an activity-driven temporal networks is in part determined by the typical activity level $a$ of its nodes. To obtain the value of ${\cal{D}}^ \ast$ for a given temporal network, we can estimate the activity probability of node $i$ in the snapshot $m$ as $a_i^m = k_i^m/k_i$, where $k_i^m$ and $k_i$ are the degree of $i$ in the snapshot and the corresponding static network. The average activity over a given snapshot $m$ with $N$ individuals is then $a^m = \mathop {\sum}\nolimits_{i = 1}^N a_i^m/N$. Then for the *j*th moment of $a$ of the whole temporal network with $M$ snapshots, we calculate it by $\langle a^j\rangle \approx \mathop {\sum}\nolimits_{m = 1}^M (a^m)^j/M$. For each of the empirical datasets we study, we find that the threshold for the outbreak of defection reaches its maximum when $\Delta t$ is at an intermediate level (Fig. 6), echoing our previous result of a Goldilocks regime of temporality maximally conducive to cooperation.

We can understand the link between the model predictions and the data by rewriting the threshold (3) as ${\cal{D}}^ \ast = \frac{2}{{1 \, + \, \sqrt {1 \, + \, {\mathrm{Var}}(a)/{\mathrm{E}}^2(a)} }}$, where ${\mathrm{Var}}(a) = \langle a^2\rangle - \langle a\rangle ^2$ and ${\mathrm{E}}(a) = \langle a\rangle$ For small $\Delta t$, individuals tend to be less active in each sparse snapshot (small ${\mathrm{E}}(a)$ implies small ${\cal{D}}^ \ast$), which in turn gives more chance for defectors to spread their strategy pairwisely as it is the Nash equilibrium. For large $\Delta t$, however, due to the heterogeneity of interactions over different snapshots (large ${\mathrm{Var}}(a)$ implies small ${\cal{D}}^ \ast$), clusters of cooperators have less chance to stabilise. Only at an intermediate $\Delta t$ can cooperators outspread defectors, and the combination of high ${\mathrm{E}}(a)$ and low ${\mathrm{Var}}(a)$ leads to a high barrier to defection (${\cal{D}}^ \ast \approx 1$) (Fig. 6).

## Discussion

We have shown that temporal networks, both empirical and synthetic, generically enhance the emergence of cooperation relative to their static counterparts. Remarkably, this central finding holds even after the underlying contact sequences are randomised, thereby destroying topological (e.g. clustering) or temporal (e.g. bursts) correlations in the data. Altogether, this suggests that temporality—and temporality alone—is sufficient to improve cooperation. Indeed, after randomisations, we find that the level of cooperation is actually improved, demonstrating that the bursty nature of human interactions hinders the maintenance of cooperation to some degree. Finally, we demonstrate that the temporality of a network determines the fate of cooperators, with cooperators flourishing at intermediate values of network temporality.

The temporal networks considered here should be contrasted with coevolutionary dynamics, in which the changes in network structure are tied to the dynamics of the relevant social dilemmas. For example, several important mechanisms elucidate that cooperation can be boosted by strategic migration^36,40^, and deliberate switching of interaction partners to avoid defectors^37,61,62^. However, it is unlikely the temporality characterising real social interactions is driven exclusively (or even primarily) by strategic switching in pursuit of a given objective^47–50^. This underscores the importance of studying cases in which the temporality is exogenous to the game dynamics, allowing an independent assessment of how the former affects the latter. As such, the agnostic view of the nature of temporality we have taken here represents a fundamental strength of our approach.

We have shown that our main conclusions are not artifacts of the specific empirical networks considered here, nor do they qualitatively change under different parameterisations. Nonetheless, future investigations such as appropriate behavioural experiments that incorporate network temporality and relevant phenomena like burstiness are warranted. Though we have shown that our results are unchanged when considering only subsets of the network (Supplementary Fig. 18), disregarding very short-lived contacts (Supplementary Fig. 19), changing the clustering coefficient (Supplementary Figs. 20 and 21), or using different starting network types (Supplementary Fig. 22), real systems display considerable variability, including different characteristic timescales for population evolution (Supplementary Fig. 23). Accordingly, future analysis of interactions at different spatial and temporal scales will be necessary to understand the full implications of temporality. Toward this end, the long tradition of combining tools from network science and statistical physics^63^ with evolutionary game theory will no doubt continue to pay dividends.

Another natural extension of the current work is to consider group interactions, which involve the interactions among individuals who are not directly connected with one another^64–66^. These interactions generate much more dynamical complexity than pairwise interactions alone^67^. In microbial populations, for example, pairwise outcomes can predict the survival of three-species competitions with accuracy as high as 90%, yet information on the outcomes of three-species competition is still needed in order to predict scenarios over larger numbers of species with high accuracy^68^. Moreover, the menu of strategies can be expanded beyond the simple dichotomy of cooperation versus defection, which represents only one axis of a broader landscape of moral behaviour^69^. For example, three-strategy games analogous to rock-paper-scissors may present a more nuanced picture of the detailed interactions characterising microbial communities, ecosystems, and human societies alike.

## Methods

### Empirical temporal networks and datasets

We construct temporal networks from empirical datasets collected by the SocioPatterns collaboration (http://www.sociopatterns.org) by aggregating contacts into undirected network links over time windows of $\Delta t$ (Fig. 1a). Thus the active time interval for the snapshot $m$ is from $(m - 1)\Delta t$ to $m\Delta t$, and a link between $i$ and $j$ exists in that snapshot if players $i$ and $j$ interact at least once in that time period (Fig. 1b). We obtain a static network in the limit where $\Delta t = {\Bbb T}$, where ${\Bbb T}$ is the last timestamp in the data, resulting in a single snapshot containing all links.

### Synthetic temporal networks

We generate temporal analogues of networks with heterogeneous or homogeneous degree distributions with specified network size $N$ and average degree $\langle k\rangle$ by first generating a base static network, using the static model^70^ and the Erdős-Rényi model^55^, respectively. We then form $M$ snapshots by randomly and independently choosing a fraction $p$ of edges to be active in each one. We have verified that our results hold under more sophisticated generative models that build temporal networks from a static network backbone, such as the activity-driven model^33^.

### Randomisations of empirical datasets

We consider four widely-used null models^29^ to randomise the empirical datasets: Randomised Edges (RE) where we randomly choose pairs of edges $(i,j)$ and $(i^{\prime},j^{\prime})$, and replace them with $(i,i^{\prime})$ and $(j,j^{\prime})$ or $(i,j^{\prime})$ and $(j,i^{\prime})$ with equal probability provided this results in no self loops; Randomly Permuted Times (RPT), where we shuffle the timestamps of the contacts, leaving their sources and targets unaltered; Randomly Permuted Times + Randomised Edges (RPTRE) which consists first of RPT followed by RE; and Time Reversal (TR), where the temporal order of the contacts is reversed.

### Reporting summary

Further information on research design is available in the Nature Research Reporting Summary linked to this article.

## Supplementary information

Supplementary Information

Reporting Summary

## Data Availability

All empirical datasets analysed in this work are publicly available through the SocioPatterns collaboration (http://www.sociopatterns.org).

## Code availability

The computer codes used in this work are available at https://github.com/leizhougetbetter/TemporalNetworks.
