# Supplementary material for: Evolution of cooperation on temporal networks
Source: Nat Commun. 2020 May 8;11:2259. doi: 10.1038/s41467-020-16088-w (PMC7210286; doi:10.1038/s41467-020-16088-w)
Supplement: Supplementary file 1 — Supplementary Information [file 41467_2020_16088_MOESM1_ESM.pdf]

Supplementary Information for

Evolution of cooperation on temporal networks

Aming Li, Lei Zhou, Qi Su, Sean P. Cornelius, Yang-Yu Liu, Long Wang & Simon A. Levin

Supplementary Note 1: Theoretical analysis

Here we provide a detailed derivation of the mean-field analysis employed in the main text to approximate the expansion of egoistic behaviour on temporal networks. Considering that each link in a temporal network typically represents an interaction initiated by one of the involved individuals, we adopt the activity-driven model of temporal networks [1]. Here we associate with each individual i a probability a_i of being active (forming links with its neighbours) in any given snapshot. When an individual becomes active, it is assumed to form links with an average of l other randomly-chosen nodes. After interactions, the average probability that a cooperator (defector) changes its strategy to defection (cooperation) is represented by λ (μ), which we can represent by the processes

$$\mathrm{C} + \mathrm{D} \xrightarrow{\lambda} 2\mathrm{D} \text{ and } \mathrm{C} + \mathrm{D} \xrightarrow{\mu} 2\mathrm{C}.$$

Considering all the three parts in equation (1), where the first term is the number of defectors from the previous snapshot m who did not change strategy; the second term represents the number of defectors that switch their strategy to cooperation; and the last two terms capture new defectors generated from cooperators in the last round; we can write the following equation relating consecutive snapshots m and $m+1$ again as

$$D_a^{m+1} = D_a^m - \mu D_a^m + (N_a - D_a^m) a l \frac{\int \mathrm{d}a' D_{a'}^m}{N} \lambda + \int \mathrm{d}a' D_{a'}^m a' l \frac{N_a - D_a^m}{N} \lambda. \tag{1}$$

In the above expression, $\int \mathrm{d}a' D_{a'}^m$, is equivalent to the total number of defectors in the snapshot m, and we denote it by D^m. By letting $Q^m = \int \mathrm{d}a' D_{a'}^m a'$, we have

$$\int \mathrm{d}a (N_a - D_a^m) a \frac{\int \mathrm{d}a' D_{a'}^m}{N} = \langle a \rangle D^m - Q^m D^m / N,$$

and

$$\int \mathrm{d}a \int \mathrm{d}a' D^m_{a'} a' \frac{N_a - D^m_a}{N} = Q^m \frac{N - D^m}{N}.$$

Hence, for equation (1), by integrating over all values of a and ignoring the second order terms (*i.e.* $Q^m D^m$ here), we obtain

$$D^{m+1} = D^m - \mu D^m + \lambda l \langle a \rangle D^m + \lambda l Q^m.$$

In terms of Q^m, on both sides of the equation (1), after multiplying a and integrating out a, we further have

$$Q^{m+1} = Q^m - \mu Q^m + \lambda l \langle a^2 \rangle D^m + \lambda l \langle a \rangle Q^m,$$

considering that

$$\int \mathrm{d}a \cdot a \cdot (N_a - D^m_a) a \frac{\int \mathrm{d}a' D^m_{a'}}{N} \approx \langle a^2 \rangle D^m,$$

and

$$\int \mathrm{d}a \cdot a \cdot \int \mathrm{d}a' D^m_{a'} a' \frac{N_a - D^m_a}{N} = Q^m \left(\langle a \rangle - \frac{D^m}{N} \right).$$

Then expressions for the dynamics of D and Q above can be written as

$$\begin{cases} \partial_m D = -\mu D + \lambda l \langle a \rangle D + \lambda l Q \\ \partial_m Q = -\mu Q + \lambda l \langle a^2 \rangle D + \lambda l \langle a \rangle Q \end{cases}.$$

By linearising the above system at the origin, we have the corresponding Jacobian matrix

$$J = \begin{pmatrix} -\mu + \lambda l \langle a \rangle & \lambda l \\ \lambda l \langle a^2 \rangle & -\mu + \lambda l \langle a \rangle \end{pmatrix},$$

with the eigenvalues

$$\lambda_{1,2} = -\mu + \lambda l \langle a \rangle \pm \lambda l \sqrt{\langle a^2 \rangle}.$$

When the larger of $\lambda_{1,2}$ is positive, we know that the origin is unstable, meaning that defectors will not die out in the whole population. Hence the threshold for instability of the no-defection equilibrium can be obtained by setting

$$-\mu + \lambda l \langle a \rangle + \lambda l \sqrt{\langle a^2 \rangle} > 0$$

which is equivalent to

$$\frac{\lambda}{\mu} > \frac{1}{l}\frac{1}{\langle a\rangle + \sqrt{\langle a^2\rangle}}.$$

In snapshot m, the average number of neighbours of the individual i is

$$k_i = la_i + l\sum_{j\neq i} a_j/(N-1),$$

where the first term is the number of links formed by i actively, and the second term captures the average number of links to i initiated from its active neighbours. Hence the average number of links for each player is

$$k = \sum k_i/N = 2l\langle a\rangle.$$

According to the evolutionary process, we know that the average probability for a defector to spread its strategy is $\lambda k = 2\lambda l\langle a\rangle$. Hence we have the following expression

$$\frac{\lambda k}{\mu} > \mathcal{D}^* = \frac{2\langle a\rangle}{\langle a\rangle + \sqrt{\langle a^2\rangle}}. \tag{2}$$

We see that the increase of defectors is determined by the magnitude of λ: the bigger the λ is, the more cooperators will switch to be defectors. Similarly, μ inhibits the expansion of defectors: the bigger μ is, the more defectors will choose to become cooperators. All told, the criteria $\lambda k/\mu \geq \mathcal{D}^*$ quantifies the difficulty for defectors to take over the whole population.

In our theoretical approximation, the average number of individuals interacting with an active individual is assumed to be l. This assumption is made based on the activity-driven model, which can represent many structural features of temporal networks [1]. The second order terms like $Q^m D^m$ are ignored in our approximation. Note that this does not affect our analysis when we linearise the corresponding system at the origin, thus has no effects on derivation of the defection threshold.

Supplementary Note 2: Further analysis

The canonical replicator equations on networks are widely used to analyse the evolution of cooperation in structured populations. However, analytical results based on replicator dynamics are only known for some special network structures where all players have the same number of neighbours [2, 3, 4, 5, 6, 7, 8]. Despite some important work dealing with heterogeneous population structures [9, 10, 11], we still lack tools to study temporal networks, the snapshots of which represent an enormous

configuration space[12, 13]. Some useful approximations exist for dynamic networks in the case of coevolution[14, 13], but here we assume the change in the network structure is independent of the game dynamics. We therefore adopt a mean-field method to analyse the spread of defectors based on epidemics spreading dynamics [1].

Nevertheless, the results obtained from the mean-filed method we employed and the approximations based on the evolutionary dynamics on networks are consistent. Indeed, by denoting the frequency of cooperators x, we can represent the relevant evolutionary dynamics as

$$\dot{x} = (1-x)f(P_C - P_D) - xf(P_D - P_C),$$

where $f(P_C - P_D)$ represents the configurational transition rate, and each strategy's payoff and frequency together determine the process [15]. For the evolutionary process of pair comparison, the Fermi function $1/[1 + e^{-\beta(P_C - P_D)}]$ from statistical mechanics is usually employed as the updating rule, which indicates the probability that a defector with payoff P_D to imitate the strategy of a cooperator with payoff P_C, and the inverse temperature $\beta \geq 0$ controls the intensity of selection [16, 17]. Taking the pair comparison as an example, we have $f(P_C - P_D) = x/[1 + e^{-\beta(P_C - P_D)}]$ and $f(P_D - P_C) = (1-x)/[1 + e^{-\beta(P_D - P_C)}]$. The above macroscopic dynamics is derived from the approximate mean value equation, and details can be found in Ref. [15].

By simplifying the function $f(\cdot)$ with μ and λ for the evolutionary process on temporal networks, we have the corresponding dynamics as

$$\dot{x} = (1-x)\mu - x\lambda.$$

The stable equilibrium of the above dynamics is $x = \mu/(\mu + \lambda)$, which yields the ultimate fraction of defectors as

$$f_D = \frac{\lambda/\mu}{1 + \lambda/\mu}. \tag{3}$$

After running simulations on temporal networks, we find that our results on the critical value of λ/μ in terms of $\mathcal{D}^*$ can provide a reliable approximation based on results given by the canonical evolutionary dynamics (Supplementary Fig. 17).

Note that implicit in the (constant) probability μ, we assume there are only μD_a^m defectors becoming cooperators from snapshot m to $m+1$. Since near the origin there are few defectors, meaning that the expected payoff of a defector is much higher than that of a cooperator. Hence the probability for a defector to become a cooperator is small, allowing us to use μD_a^m for the

approximation.

Indeed, when we also consider the net conversion of defectors in snapshot m to cooperators in snapshot $m+1$, we know that it consists of two parts. The first part captures an active defector interacting with a neighbouring cooperator, and then imitating its strategy, and it can be depicted by

$$D_a^m al \frac{\int \mathrm{d}a'(N_{a'} - D_{a'}^m)}{N}\mu. \tag{4}$$

The second part tells a defector interacting with its active neighbouring cooperator, and can be expressed as

$$\int \mathrm{d}a'(N_{a'} - D_{a'}^m)a'l\frac{D_a^m}{N}\mu. \tag{5}$$

By combining equations (4) and (5), we can now re-express equation (1) as

$$\begin{aligned} D_a^{m+1} = D_a^m + (N_a - D_a^m)al\frac{\int \mathrm{d}a' D_{a'}^m}{N}\lambda + \int \mathrm{d}a' D_{a'}^m a'l \frac{N_a - D_a^m}{N}\lambda \\ - D_a^m al\frac{\int \mathrm{d}a'(N_{a'} - D_{a'}^m)}{N}\mu - \int \mathrm{d}a'(N_{a'} - D_{a'}^m)a'l\frac{D_a^m}{N}\mu, \end{aligned} \tag{6}$$

which further gives

$$D^{m+1} = D^m + \lambda l\langle a\rangle D^m + \lambda l Q^m - \mu l\langle a\rangle D^m - \mu l Q^m,$$

and

$$Q^{m+1} = Q^m + \lambda l\langle a^2\rangle D^m + \lambda l\langle a\rangle Q^m - \mu l\langle a\rangle Q^m.$$

Hence we obtain the corresponding Jacobian matrix

$$J = \begin{pmatrix} \lambda l\langle a\rangle - \langle a\rangle\mu l & \lambda l - \mu l \\ \lambda l\langle a^2\rangle & \lambda l\langle a\rangle - \mu l\langle a\rangle \end{pmatrix},$$

from which we can see that the maximum eigenvalue of J will not change when μ is small, yielding the same conclusion as shown in expression (2).

Supplementary Table

Supplemental Table 1: Statistics of the datasets. The four datasets we employed are interactions between: attendees of a ACM Hypertext conference over about 2.5 days from 8am on Jun. 29th 2009 (ACM conference), students in 5 classes at a high school in Marseilles, France over a period of 7 days in Nov. 2012 (Student 2012), in 9 classes at a high school in Marseilles, France over 5 days in Dec. 2013 (Student 2013), individuals in an office building in France, from Jun. 24 to Jul. 5, 2013 (Office 2013). The number of snapshots is calculated based on the total time window $\mathbb{T}$ over which the data were collected, and Δt (in seconds) is the time window used to aggregate the contacts into snapshots. Contacts are defined as individual triples (t, i, j) in the data, meaning nodes i and j were observed interacting in the time interval $(t, t + 20\text{s}]$. Events (links), on the other hand, are continuous interactions formed by coalescing time-adjacent contacts between the same i and j.

	ACM conference	Student 2012	Student 2013	Office 2013
Number of nodes	113	180	327	92
Number of snapshots	$\lceil 212,360\text{s}/\Delta t \rceil$	$\lceil 729,520\text{s}/\Delta t \rceil$	$\lceil 363,580\text{s}/\Delta t \rceil$	$\lceil 987,640\text{s}/\Delta t \rceil$
Number of contacts	20,808	45,047	188,508	9,827
Number of events (links)	9,865	19,774	67,613	4,592
Recording period (day)	2.5	7	5	12
Time resolution (second)	20	20	20	20

Supplementary Figures

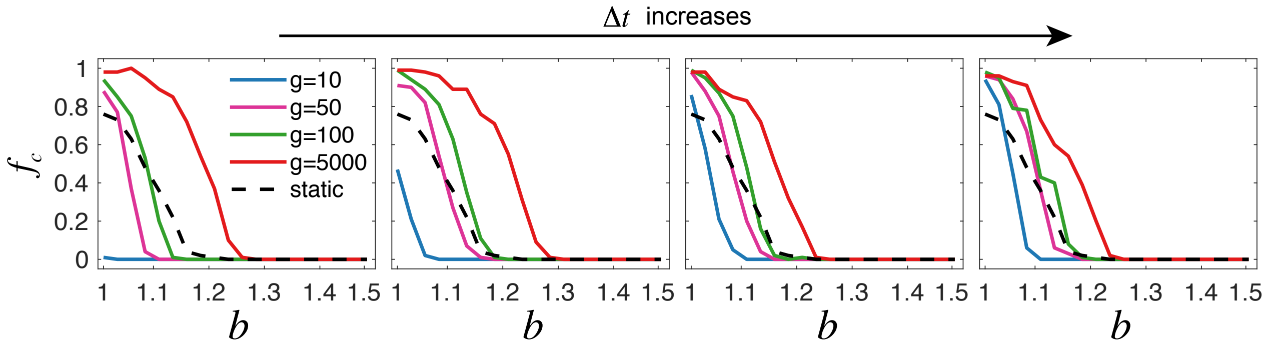

Supplemental Figure 1: Evolution of cooperation on temporal networks with the original time scale of the dataset. By constructing snapshots under a given time window, we might lose some information regarding the heterogeneity of individual interactions on temporal networks. Here we adopt the original time scale of network edges to study the evolution of cooperation on temporal networks defined by the dataset Student 2013. We find that our main conclusions presented in Fig. 2 still hold when we consider the different time scales. Indeed, for each edge given in the dataset, the corresponding individuals will interact with each other successively, and it is possible now for the link existed in each snapshot to capture several times of interactions. After employing Δt to indicate the time period over which individuals will update their strategies, equivalently we have the evolution of cooperation over the weighted snapshot, where the link weights define how many times the corresponding pair of individuals will interact. For static networks (where Δt is big enough to aggregate all data into a weighted snapshot), our settings are similar to the evolution of cooperation on traditional weighted static networks. This confirms that our results are robust for weighted snapshots, where interactions are allowed to happen diversely. Other parameters are the same as those in Fig. 2, and results shown here are also applicable for other datasets.

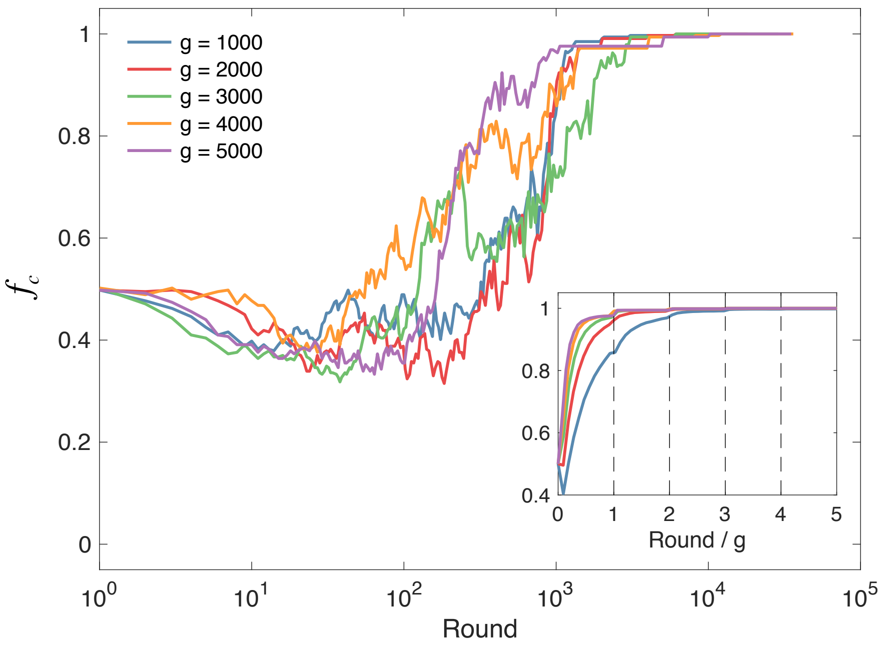

Supplemental Figure 2: Temporal evolution of the fraction of cooperators. Here we show one realisation of temporal dynamics of the fraction of cooperators ($f_{\rm c}$) under a fixed b as a function of time (game round). In the inset, the horizontal axis indicates the normalised time scale for different g, we demonstrate that $f_{\rm c}$ tends to increase when the snapshot switches (indicated by vertical dashed lines) irrespective of the value of g. This points to the existence of multilevel selection [18] and the evolution of network snapshots facilitates the increment of $f_{\rm c}$. Lines in the inset are averaged over 500 realisations. Here $b = 1.11$ with $\Delta t = 6$ hr for the dataset Student 2013.

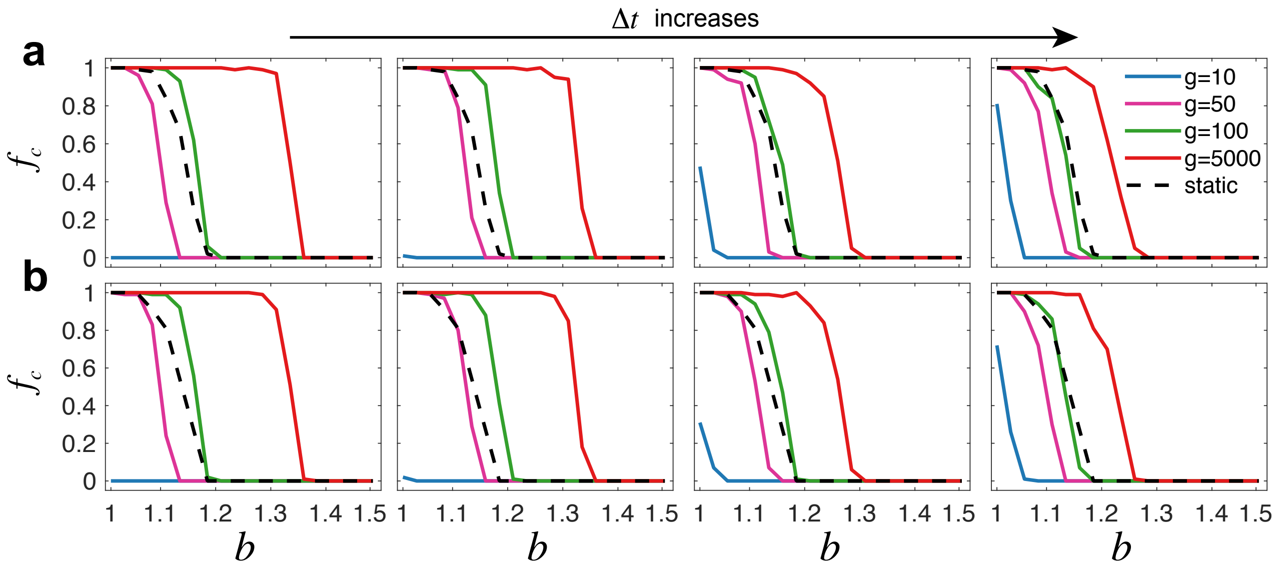

Supplemental Figure 3: Evolution of cooperation on temporal networks with asynchronous updating schemes. For each snapshot, we have shown that temporal networks can facilitate the evolution of cooperation, where at the end of each round, players update their strategies in synchrony. Here we consider the scenario where at the end of each round, only a fraction of players (p) choose to update strategies [19], and $p = 0.2, 0.5$ for panels (**a**) and (**b**), respectively. We find that the asynchronous updating scheme does not bring any qualitative modifications to our results reported in Fig. 2. For the dataset Student 2013, the results are presented in the same style of Fig. 2, and g represents the averaged updating times for each player. Other parameters are the same as those in Fig. 2, and results shown here are also applicable for other datasets.

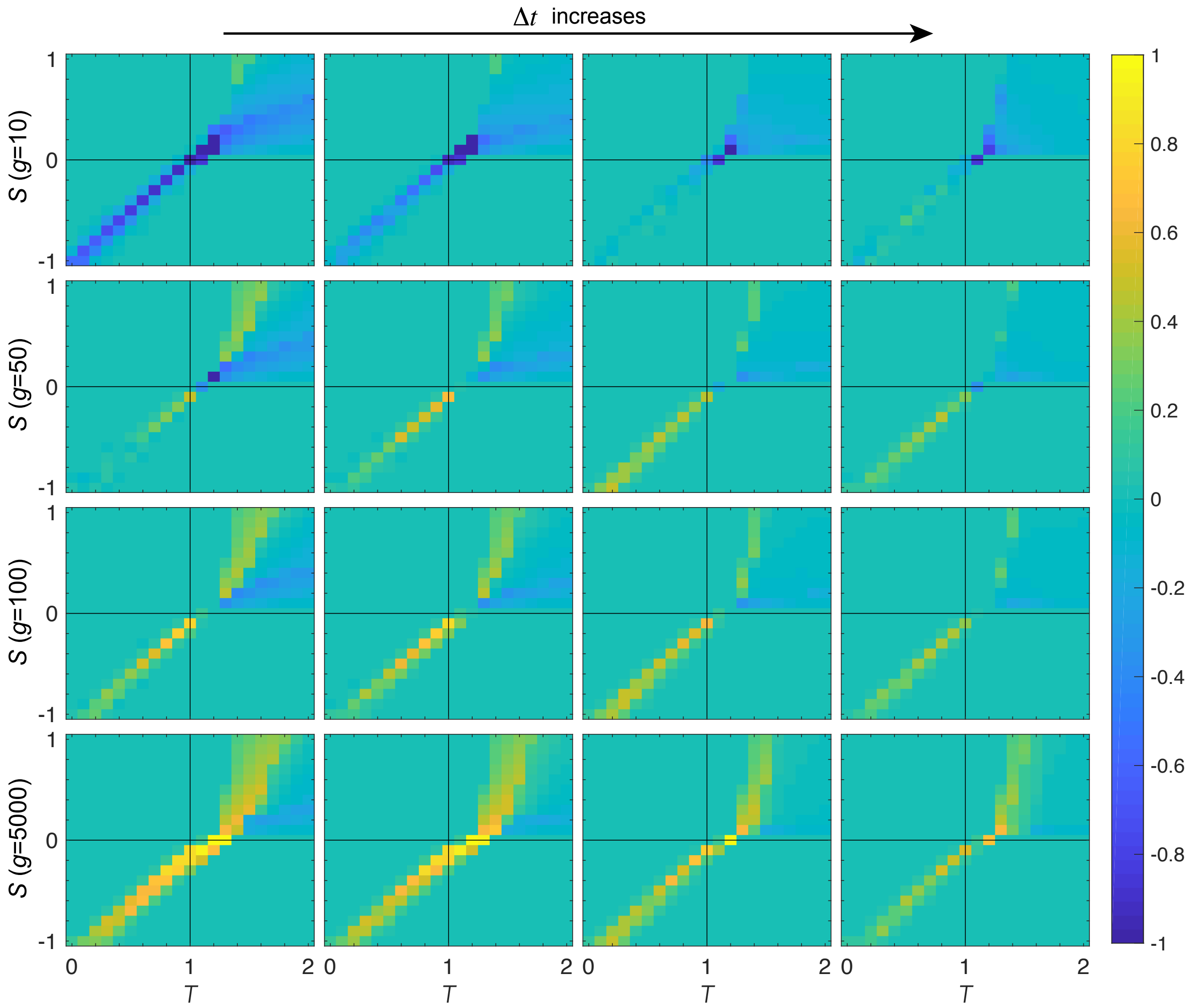

Supplemental Figure 4: Evolution of cooperation on temporal networks under different social dilemmas. We now consider the general social dilemmas with two parameters. Indeed, the general four parameters R, S, T and P presented in the main text can be conventionally reduced to two (*i.e.* S and T) by normalising the difference between mutual cooperation (R) and defection (P) to 1 (*i.e.* $R = 1$ and $P = 0$) [20]. As a function of $-1 < S < 1$ and $0 < T < 2$, we plot the frequency of cooperators as a contour for the dataset Student 2013 with different Δt. The colour represents the frequency of cooperators of temporal networks minus that of the static networks. In each panel, the lower triangle in the upper-right quadrant represents the Snowdrift game ($S \geq 0, T > 1$ and $T + S < 2$); lower-left quadrant ($S < 0$ and $T \leq 1$) indicates the Stag-Hunt game, and lower-right quadrant ($S < 0$ and $P > 1$) indicates the Prisoner's Dilemma. Under other two social dilemmas (Stag-Hunt game, and Snowdrift game) and the Prisoners Dilemma with other values of S, we find that temporal networks can still favour the evolution of cooperation compared to the corresponding static networks (*i.e.*, the difference of the frequency of cooperators is still positive for middle value of Δt and large g). Here we validate that our results are applicable for other types of social dilemmas. Other parameters are the same as those in Fig. 2, and results shown here are also applicable for other datasets.

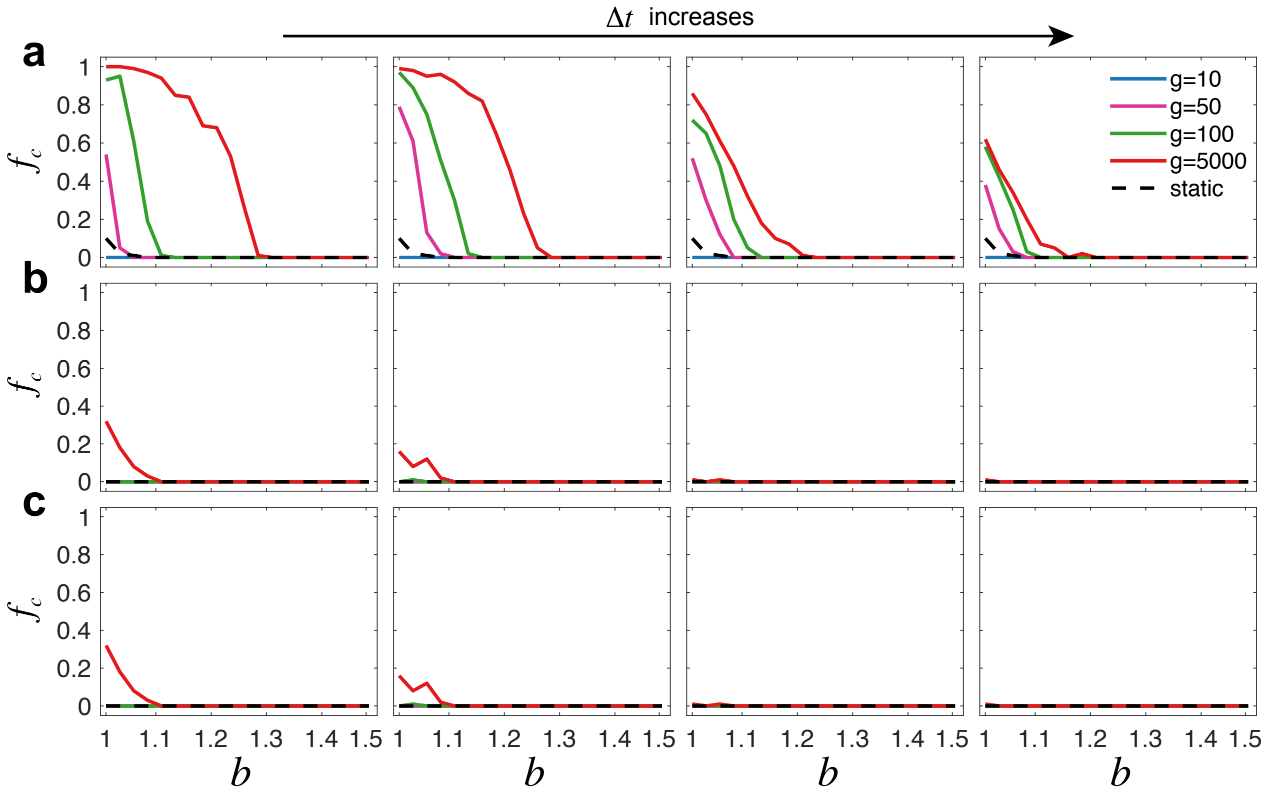

Supplemental Figure 5: Evolution of cooperation on temporal networks under the Prisoner's Dilemma. For the social dilemma we have studied in Fig. 2, it is the (weak) Prisoner's Dilemma with $S = 0$ [21, 22, 23], and this case allows us to focus on a single parameter b (T) to explore the effects of temporal networks on the evolution of cooperation. We now consider the general case of the Prisoner's dilemma where $S \neq 0$. Similar to Fig. 2c on the dataset Student 2013, we plot the frequency of cooperators as a function of different b for $S = -0.1$ (panel (**a**)), $S = -0.3$ (panel (**b**)), and $S = -0.7$ (panel (**c**)). We find that temporal networks can still favour the evolution of cooperation compared to static networks when S is close to 0. With the decrease of S, cooperators almost die out on temporal and static networks due to the high dilemma, nevertheless we still can find situations where temporal networks can be relatively better than static networks for facilitating cooperation. This confirms that our results are robust for the general case of the Prisoner's Dilemma with $S \neq 0$. Other parameters are the same as those in Fig. 2, and results shown here are also applicable for other datasets. For other values of S and T, please see Supplementary Fig. 4.

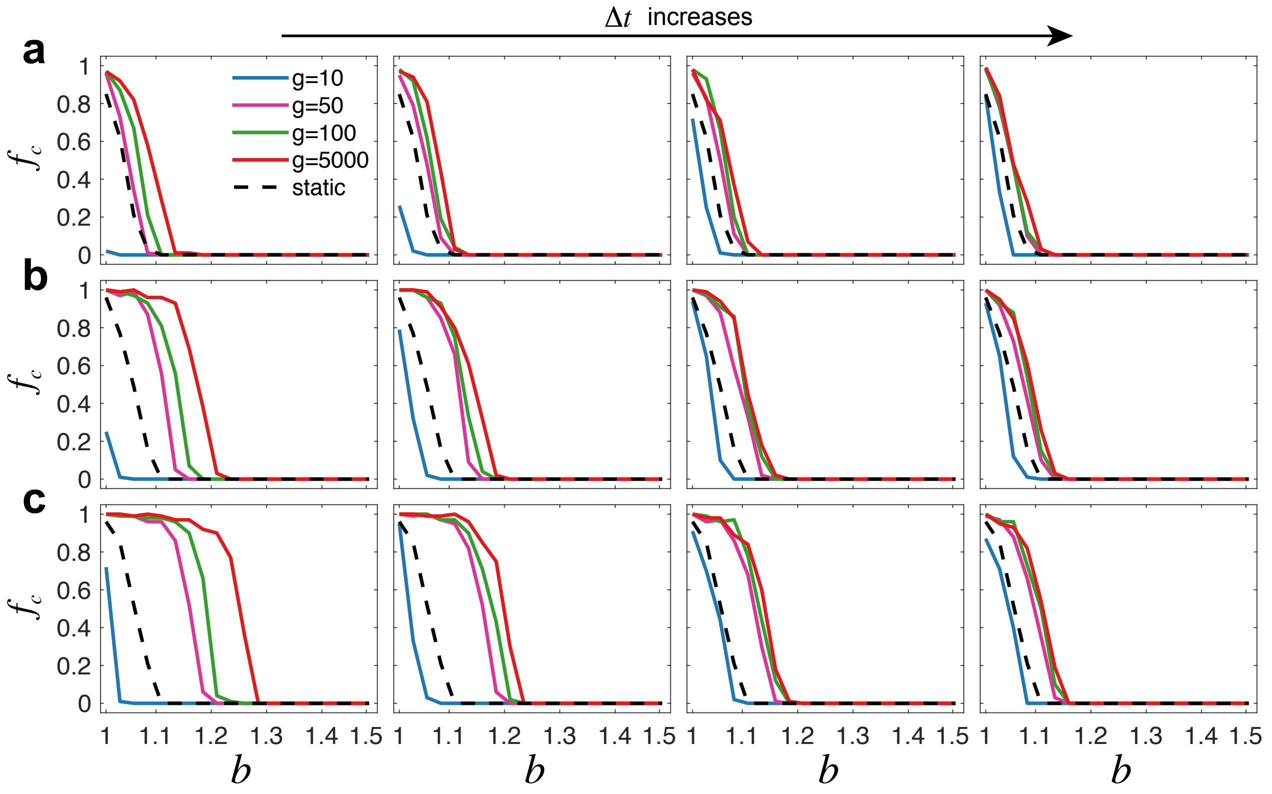

Supplemental Figure 6: Evolution of cooperation on temporal networks with pairwise comparison. For the rule we have studied in Fig. 2, it is the imitation dynamics, where individuals only imitate more successful ones. Here we show the results under the evolutionary process with the so-called pairwise comparison where individuals can imitate strategies that perform worse [16]. Specifically, the ubiquitous Fermi distribution function from statistical mechanics is used to represent the probability $p = 1 \big/ \left[1 + \mathrm{e}^{-\beta(P_j - P_i)}\right]$ that i (with payoff P_i) imitates j (with payoff P_j). The inverse temperature controls the intensity of selection β in this process [16, 24]. In pairwise comparison, it gives the possibility for less successful individual to replace the more successful one (especially for $\beta \to 0$). Here we validate that the switching from the imitation dynamics to pairwise comparison does not bring any qualitative modifications to our previous results. For the dataset Student 2013, panels (**a**), (**b**), and (**c**) correspond to $\beta = 0.5, 1,$ and 10, respectively. Other parameters are the same as those in Fig. 2, and results shown here are also applicable for other datasets.

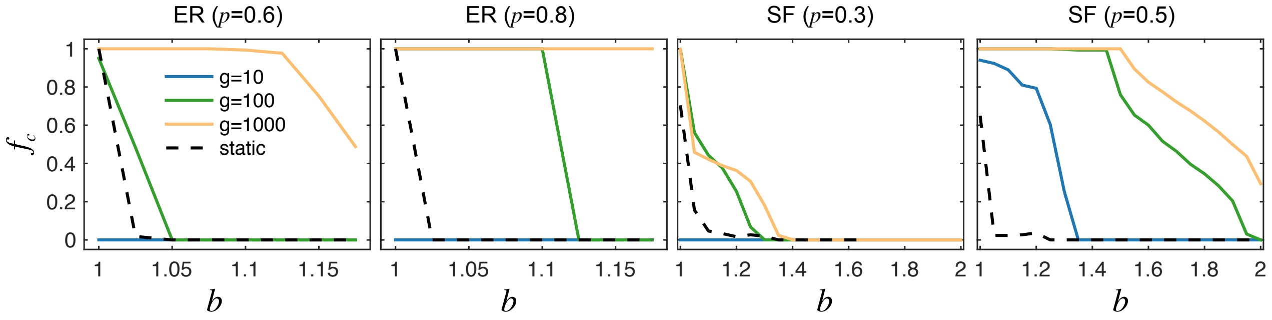

Supplemental Figure 7: Evolution of cooperation on synthetic temporal networks. Here we generate M sparse snapshots based on M different scale-free networks with preferential attachment [25] and Erdős-Rényi random networks [26] with the network size $N = 1000$ and average degree $\langle k \rangle = 4$ (see Methods). Our results shown in Fig. 3 are also validated with different p, which determines the level of link activity of each snapshot. Other parameters are the same as those in Fig. 3.

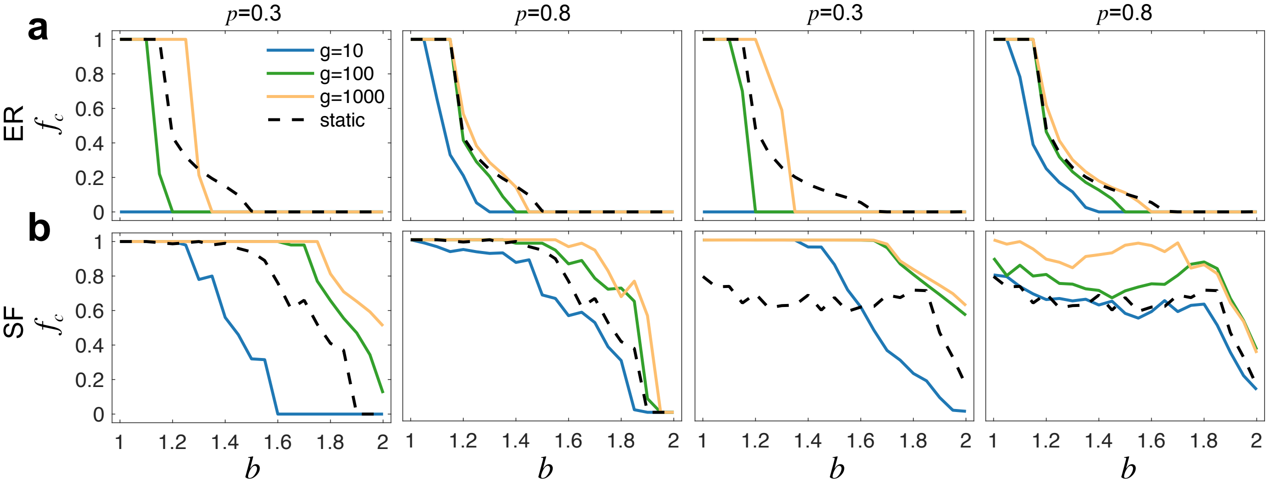

Supplemental Figure 8: Evolution of cooperation on synthetic temporal networks with different network sizes. For different network sizes, here we present the frequency of cooperators for the base Erdős-Rényi random (**a**) and scale-free (**b**) networks. For first two columns, $N = 600$, and last two columns correspond to $N = 1500$. Other parameters are the same as those in Fig. 3.

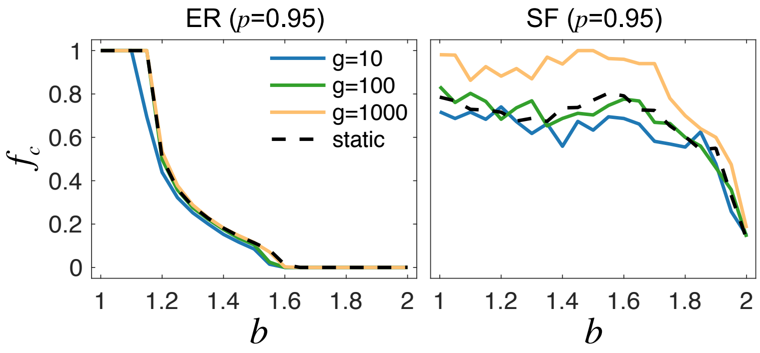

Supplemental Figure 9: Evolution of cooperation on synthetic temporal networks. Similar to the results shown in Fig. 3, here we choose $p = 0.95$. There are more links being active in each snapshot when p is big, which reduces the gap of the results obtained from temporal and static networks. Other parameters are the same as those in Fig. 3.

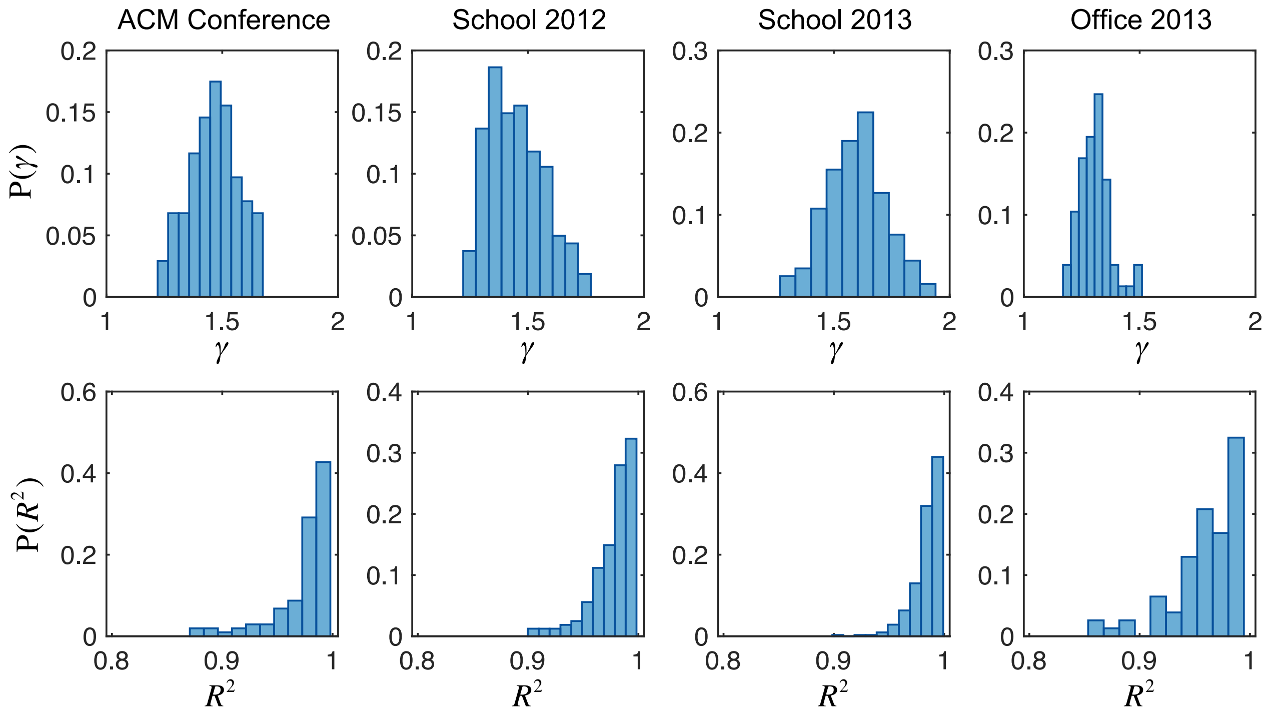

Supplemental Figure 10: Bursty behaviour in four datasets. For every dataset, we obtain a number of inter-event time τ for each individual based on his or her interactive logs. For an individual, as the number is bigger than 30, we fit all τ with power-law distribution $P(\tau) \sim \tau^{-\gamma}$, generating a γ and an adjusted R^2. For each dataset, we give the distributions of γ and the adjusted R^2 for all individuals there.

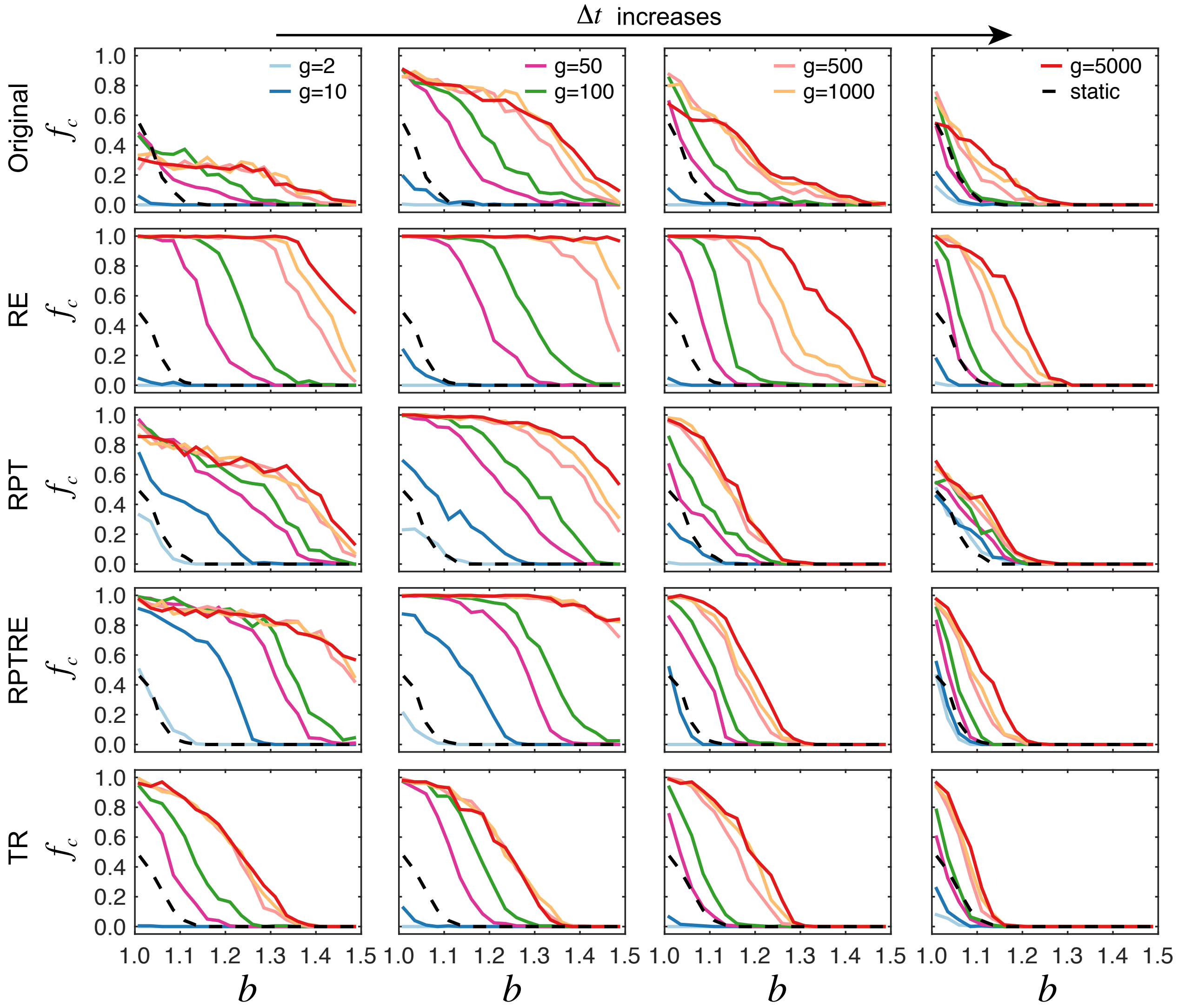

Supplemental Figure 11: Evolution of cooperation on temporal networks generated from the original and randomised ACM conference dataset. For different null models, we show the fraction of cooperators f_c as a function of the dilemma parameter b for different g. RE and TR have no effect on the correlations in temporal activity by construction, and hence have no effects on network temporality apparently. RPT and RPTRE, on the other hand, destroy the temporal correlations between edges, thereby lowering the (too high) temporality of the system. Thus for small g under RPT and RPTRE, f_c is increased markedly relative to the original dataset, while for large g the gains are more modest. The above findings are also true for other datasets (see Figs. 12 to 14). Overall, our results showing that temporal networks could facilitate the evolution of cooperation are robust even after the data is randomised. Other parameters are the same as those in Fig. 2.

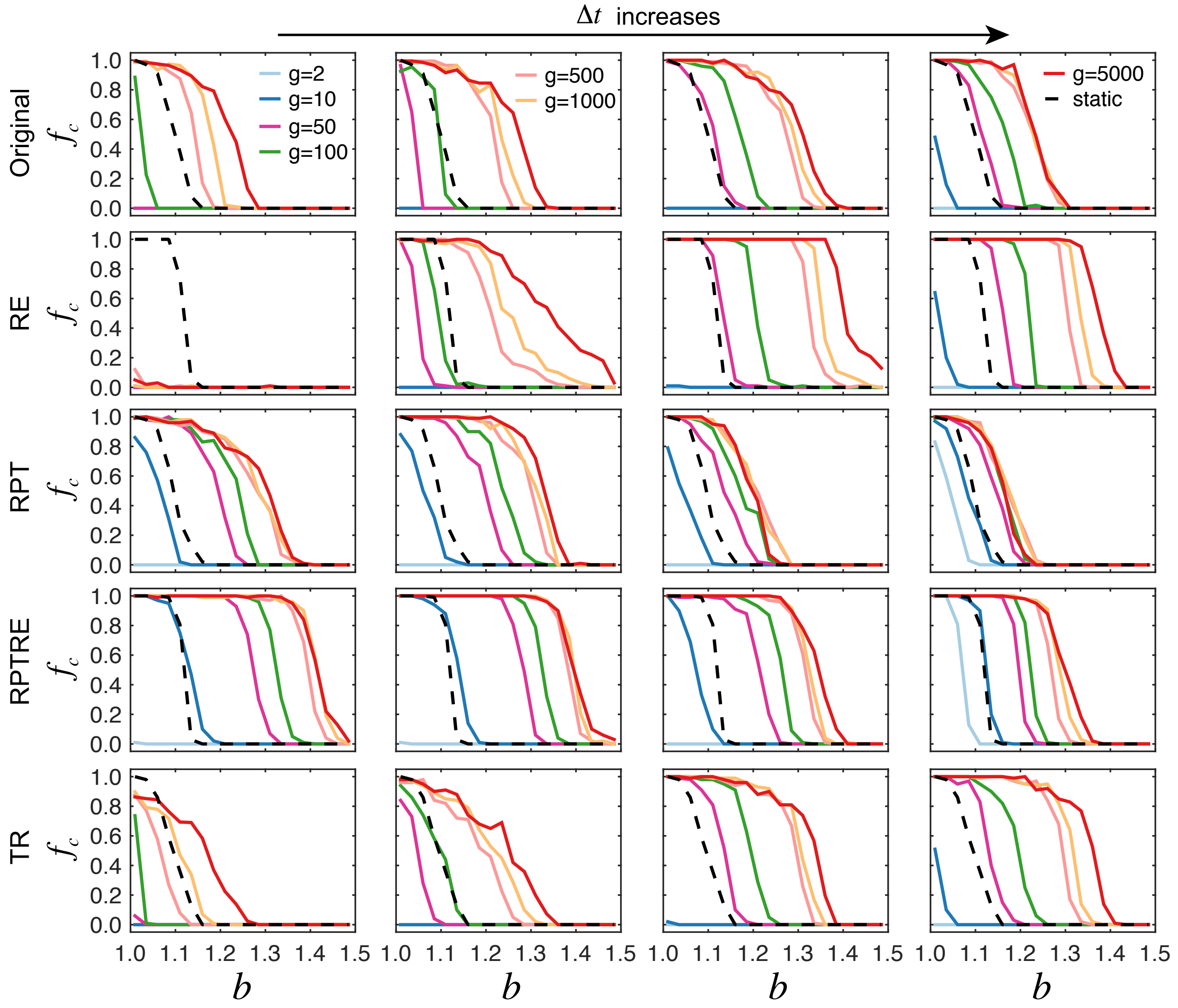

Supplemental Figure 12: Evolution of cooperation on temporal networks generated from the original and randomised Student 2012 dataset. Note that when Δt is small, Randomised Edges (RE) has the effect of breaking up the network structure within the (already sparse) snapshots, inhibiting cooperation. Likewise, when g is big, RPT fails to improve f_c either owing to the small resulting temporality. All parameters are the same as those in Fig. 2.

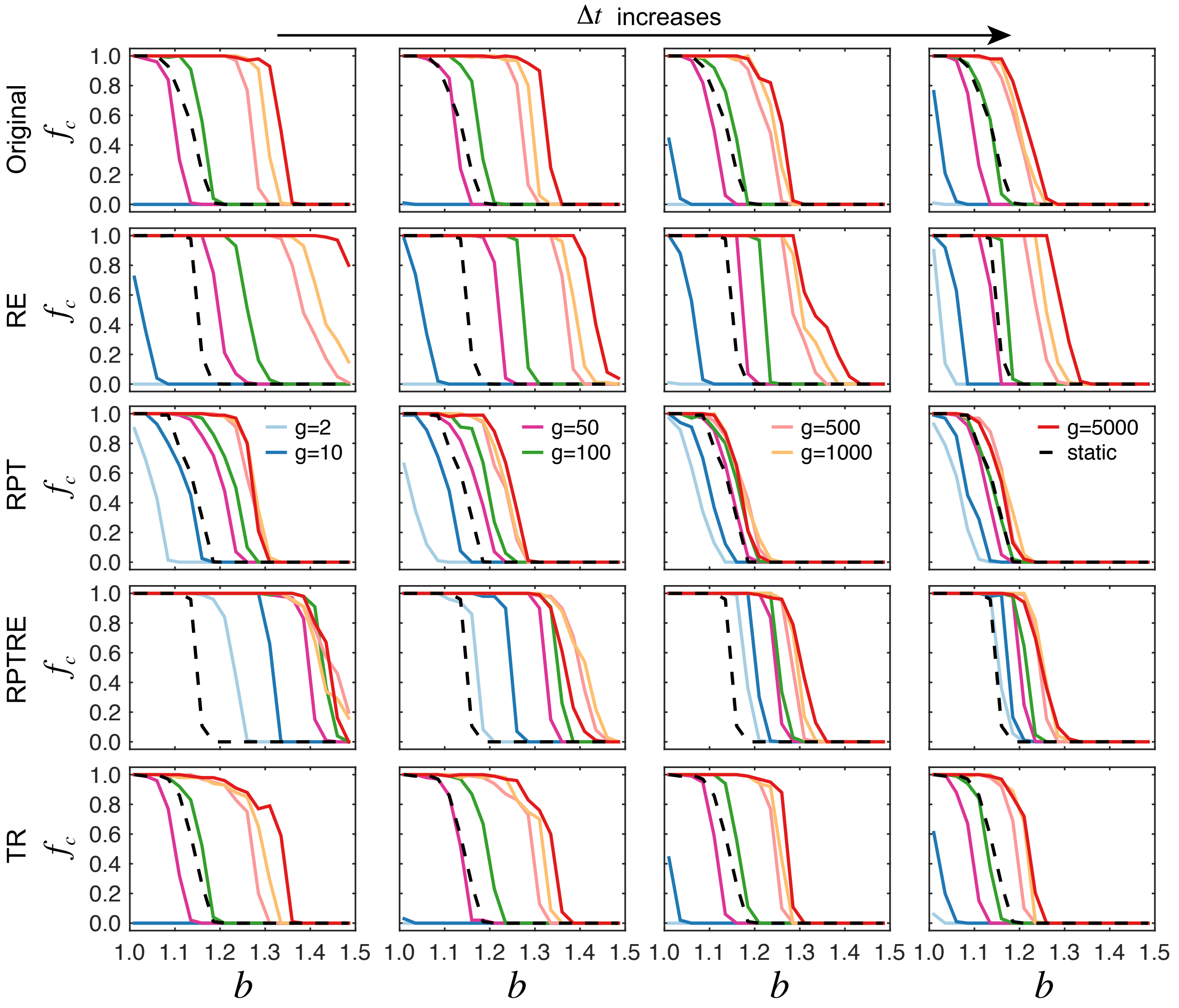

Supplemental Figure 13: Evolution of cooperation on temporal networks generated from the original and randomised Student 2013 dataset. All parameters are the same as those in Fig. 2.

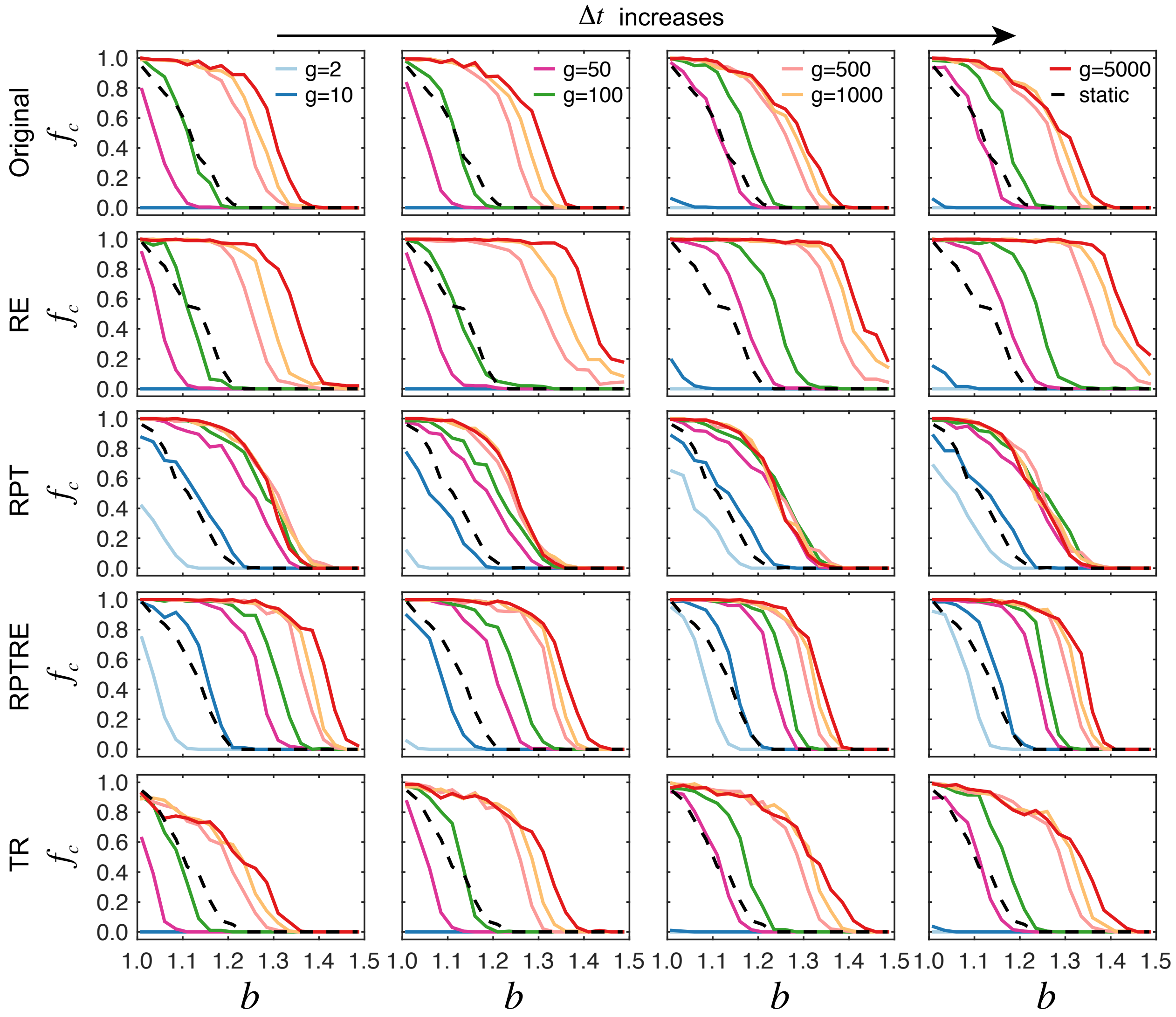

Supplemental Figure 14: Evolution of cooperation on temporal networks generated from the original and randomised Office 2013 dataset. All parameters are the same as those in Fig. 2.

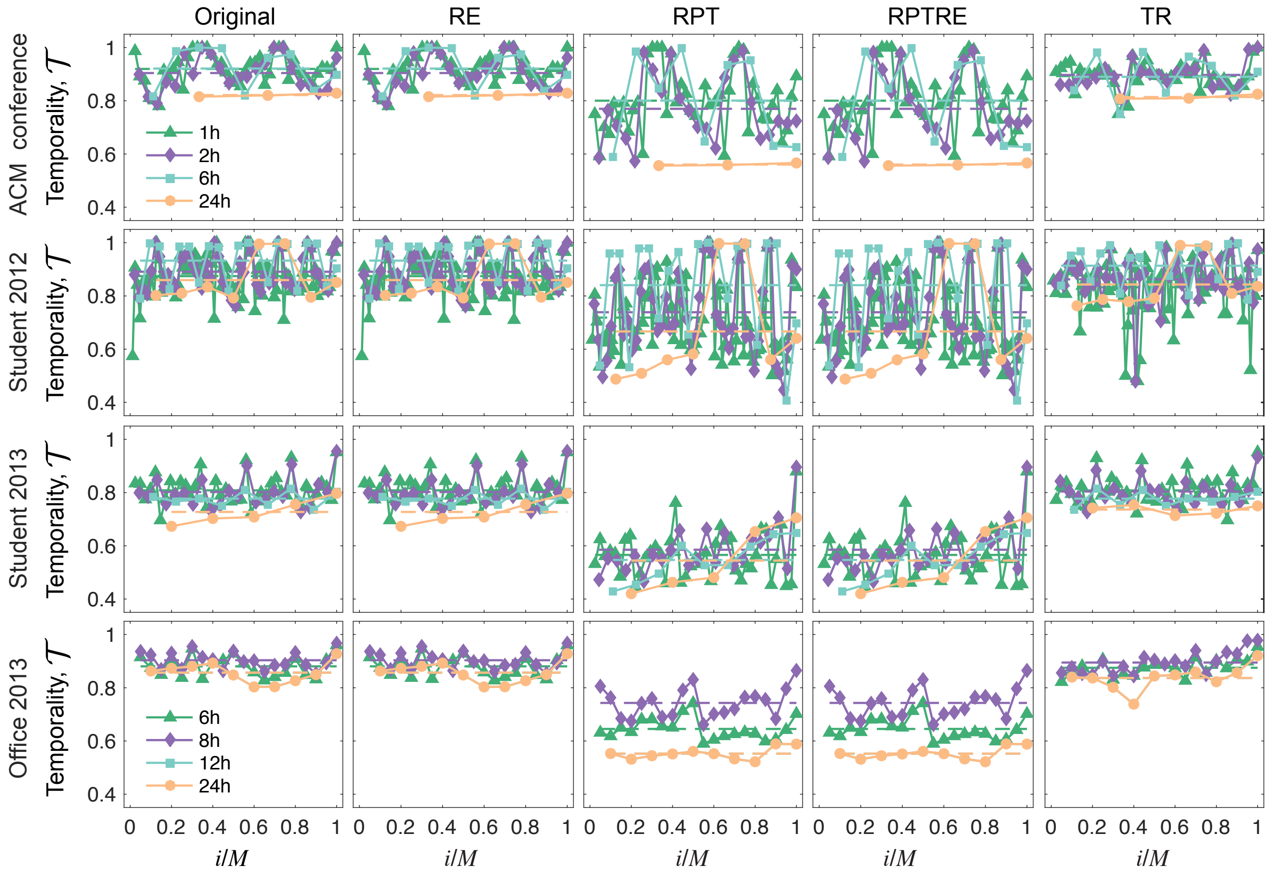

Supplemental Figure 15: How temporality arises from differences between successive snapshots. Each time point shows the contribution to the temporality as defined in the main text made by each pair of snapshots m and $m+1$. The total temporality $\mathcal{T}$ is the average of these contributions. Randomisations that destroy temporal correlations in nodal activity (RPT, RPTRE) have the effect of lowering this average. For every curve, we normalise the index of each snapshot under different Δt by dividing the corresponding number of snapshots.

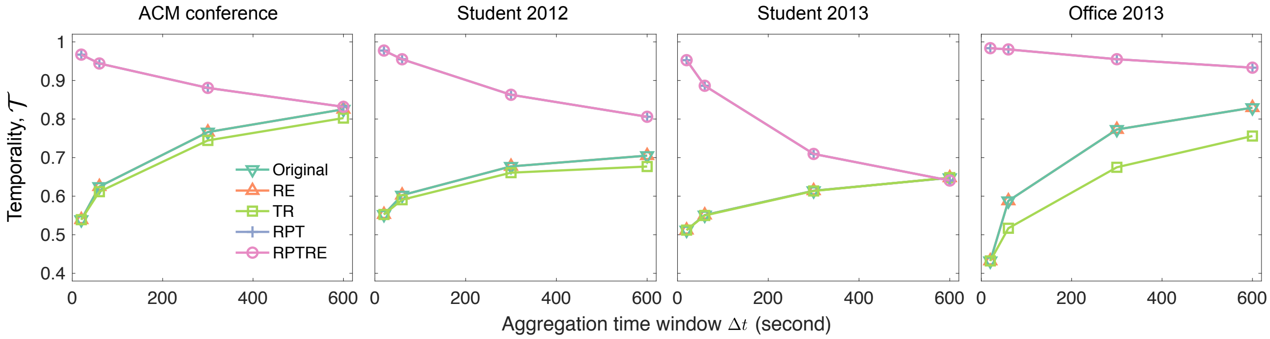

Supplemental Figure 16: Effect of time window on network temporality. For the datasets in our study, we plot the temporality $\mathcal{T}$ as a function of aggregation time window Δt, which aggregates the original data (lower triangle line) into snapshots of different in the short limit. We see that $\mathcal{T}$ depends on Δt, and specifically that more granular snapshots (low Δt) implies less predictability ($\mathcal{T}$ close to 0.5). This should be contrasted with the longer Δt (= 3600 seconds) used in Fig. 5 of the main text. Note that in the very small Δt regime considered here, cooperation cannot be built, and hence our explorations in Fig. 5 of the main text focus on the longer time regime. We have also shown $\mathcal{T}$ after randomising the data following the various schemes used in the main text.

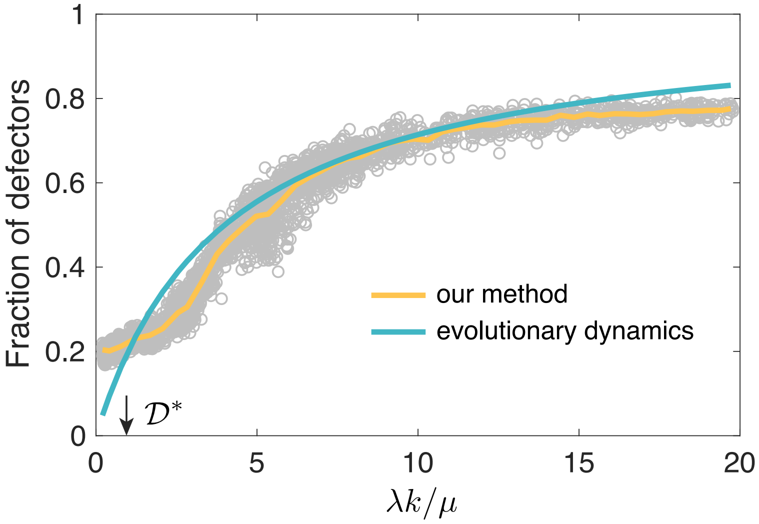

Supplemental Figure 17: Numerical validation of the theoretical approximation on the spread of defectors. For each realisation of the simulation, we choose (independently and uniformly at random) the values of $-1 < S < 1$ and $0 < T < 2$ with $R = 1$ and $P = 0$, and then calculate the average probability λ (μ) of a cooperator (defector) changing to be defector (cooperator). Each grey circle represents one realisation with $k\lambda/\mu$ averaged over whole process and the corresponding final fraction of defectors. The gold line indicates the average trend over all $4,000$ simulations, and the blue line captures the analytical fraction of defectors based on equation (3). To intuitively show the threshold in the equation (2), we plot the fraction of defectors as a function of $\lambda k/\mu$, where $k = 2l\langle a \rangle$. The arrow indicates the threshold $\mathcal{D}^*$, where we can see that above $\lambda k/\mu > \mathcal{D}^*$, the fraction of defectors increases rapidly away from 0. Here the temporal network is generated according to the activity-driven model with $1,000$ nodes, $l = 4$, and a is selected independently and uniformly at random between 0 and 1 for each node [1]. The updating rule follows the pairwise comparison under the Fermi function with $\beta = 10$ [16]. The robustness of these results has been checked over different choices of parameters with different network sizes, updating rules, and asynchronous updating process, and no qualitative changes are observed.

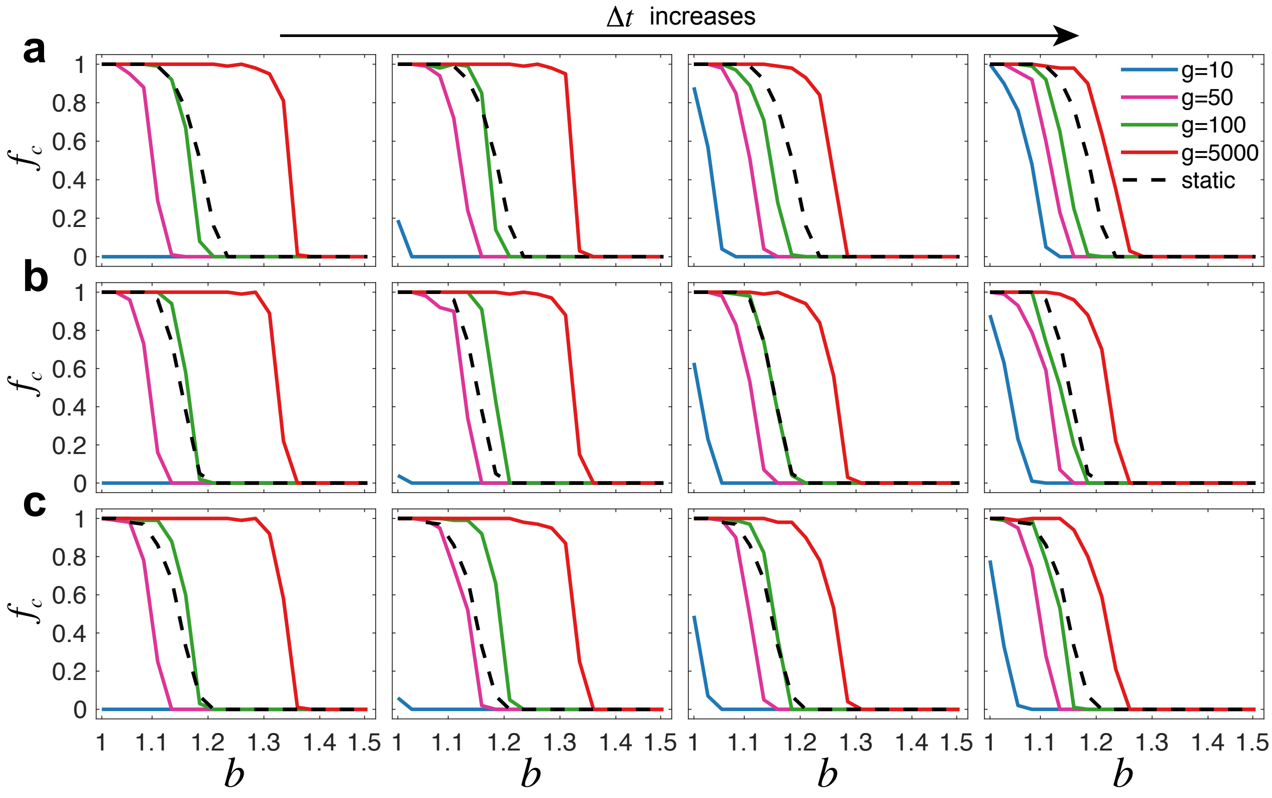

Supplemental Figure 18: Evolution of cooperation on temporal networks constructed from partial empirical data. For the dataset Student 2013 collected over five days, we here study the evolution of cooperation on temporal networks constructed from only the first two (panel (**a**)), three (panel (**b**)), or four (panel (**c**)) days. We find that results obtained from partial empirical data do not bring any qualitative modifications to our main results reported in Fig. 2. Other parameters are the same as those in Fig. 2, and the results here are representative of the other datasets.

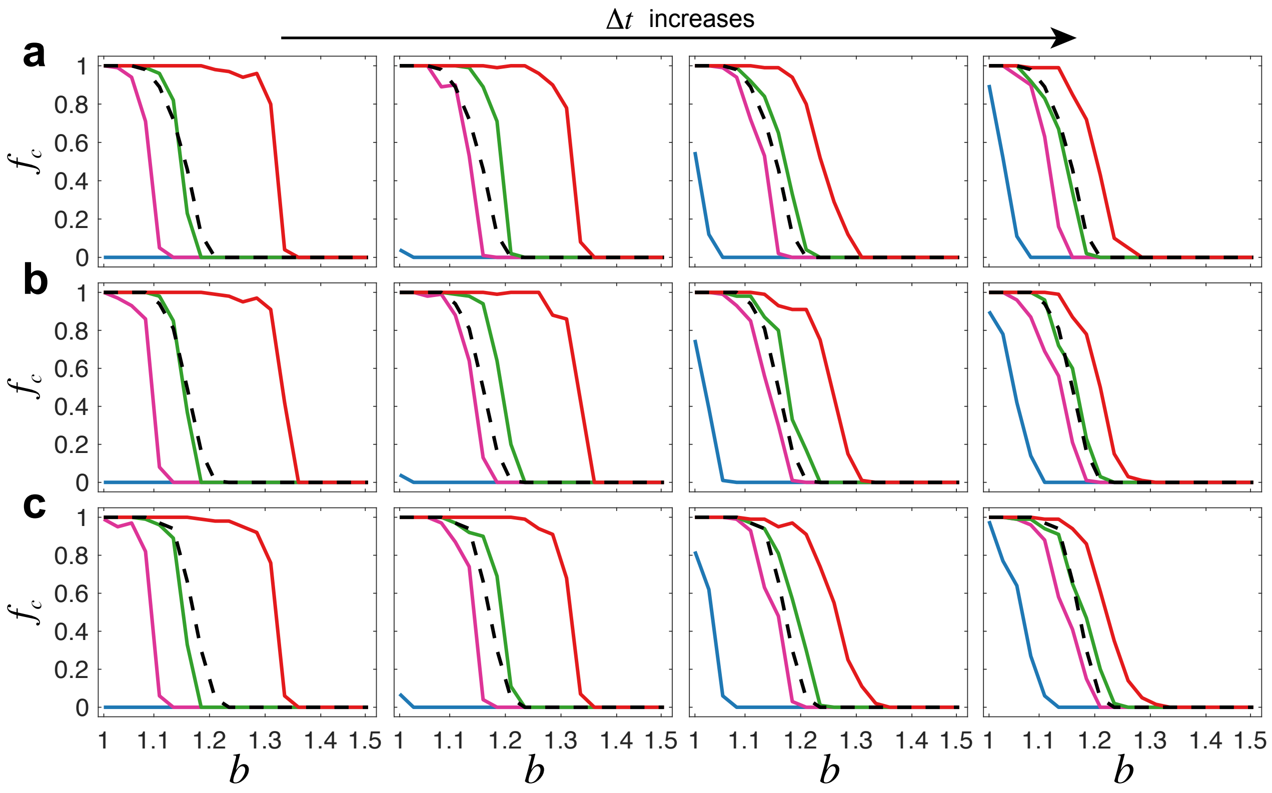

Supplemental Figure 19: Evolution of cooperation on temporal networks constructed from filtered empirical data. For the dataset Student 2013, here we first exclude all contacts lasting less than 20 (panel (**a**)), 40 (panel (**b**)) or 60 (panel (**c**)) seconds, and then construct the corresponding temporal networks. We find that disregarding short-lived contacts in this way does not bring any qualitative modifications to our main results reported in Fig. 2. Other parameters are the same as those in Fig. 2, and results shown here are also applicable for other datasets.

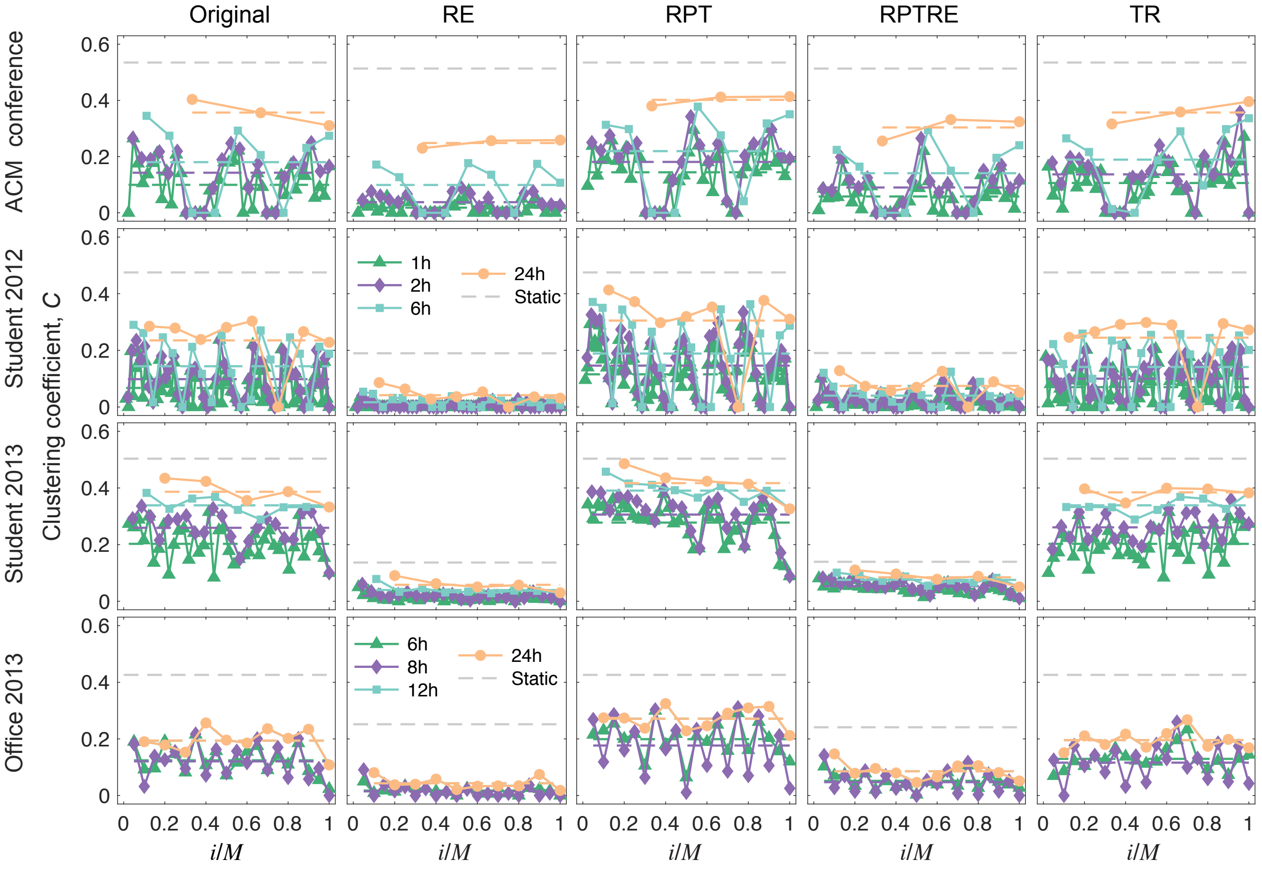

Supplemental Figure 20: Illustration on the clustering coefficient over each snapshot. Each time point shows the clustering coefficient C of the corresponding snapshot under different Δt, as defined in previous work [27]. For comparison, we also show the average over all snapshots, which is indicated by the horizontal line with the same colour. The clustering coefficient for the corresponding static network is presented by the grey line in each panel. For every curve, we normalise the index of each snapshot under different Δt by dividing the corresponding number of snapshots. All other parameters are the same as those in Fig. 2.

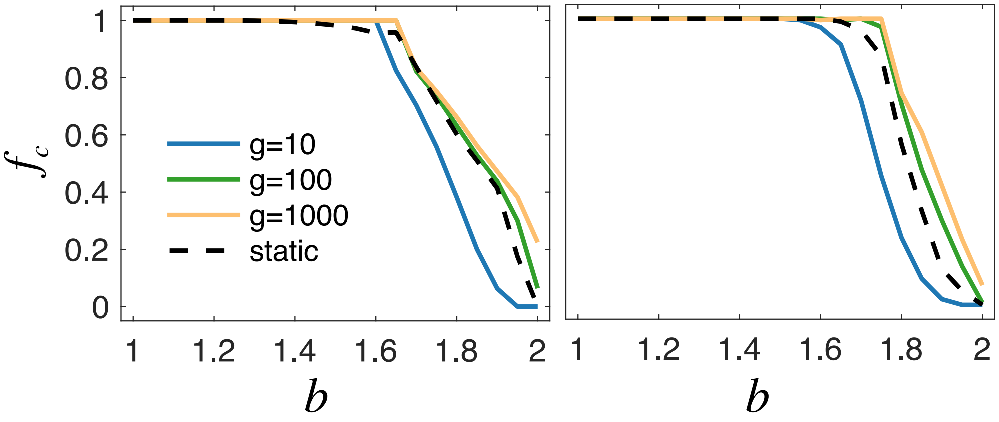

Supplemental Figure 21: Evolution of cooperation on synthetic temporal networks with different clustering coefficients. Here we simulate the game dynamics on a temporal network generated from a base scale-free (SF) network with different clustering coefficients under Holme-Kim model [28]. For (a) and (b), the clustering coefficient is 0.038 and 0.36, respectively. Here we set $p = 0.8$, and other parameters are the same as those in Fig. 3.

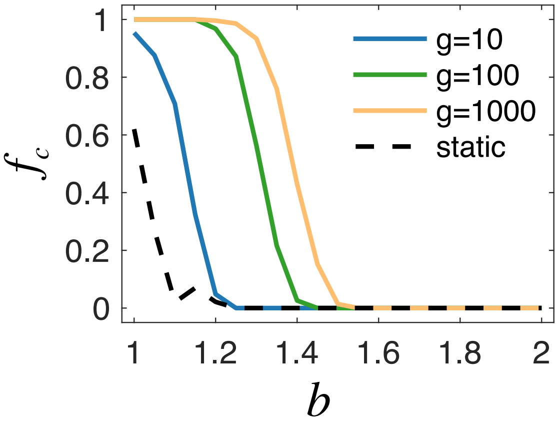

Supplemental Figure 22: Evolution of cooperation on synthetic temporal networks with different starting network types. Here we generate M sparse snapshots using $M/2$ different scale-free (SF) networks with preferential attachment [25] and $M/2$ Erdős-Rényi (ER) random networks [26]. The order of each snapshot based on SF or ER network is assigned randomly. Other parameters are the same as those in the last panel of Fig. 7.

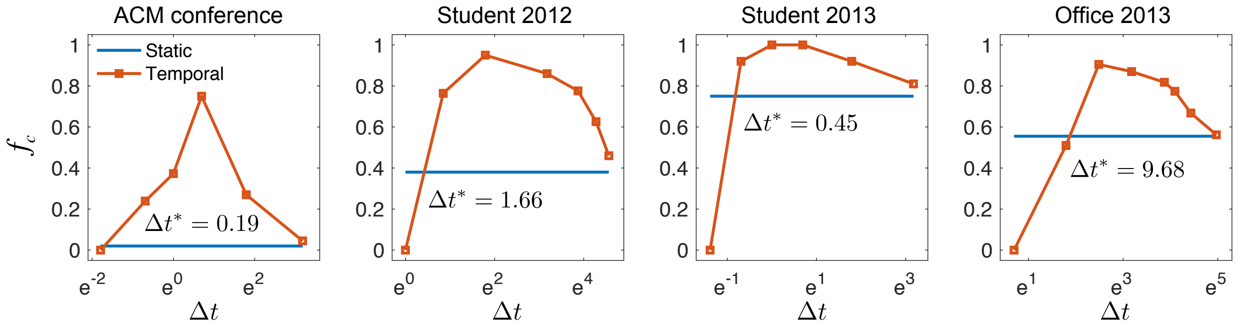

Supplemental Figure 23: Cooperation as a function of aggregation time window for the empirical networks. For each dataset, we plot the fraction of cooperators (f_c) over different time windows (Δt). The cooperation level for the corresponding static networks is shown by the blue horizontal lines. In each panel, we note the minimum value of Δt (indicated by Δt^*), at which temporal networks overtake static networks. Here $b = 1.11$, $g = 100$, and other parameters are the same as those in Fig. 2.

Supplementary References

[1] Perra, N., Gonçalves, B., Pastor-Satorras, R. & Vespignani, A. Activity driven modeling of time varying networks. *Sci. Rep.* **4**, 469 (2012).

[2] Hauert, C. & Doebeli, M. Spatial structure often inhibits the evolution of cooperation in the snowdrift game. *Nature* **428**, 643–646 (2004).

[3] Ohtsuki, H., Hauert, C., Lieberman, E. & Nowak, M. A simple rule for the evolution of cooperation on graphs and social networks. *Nature* **441**, 502–505 (2006).

[4] Ohtsuki, H. & Nowak, M. A. The replicator equation on graphs. *J. Theor. Bio.* **243**, 86–97 (2006).

[5] Taylor, P. D., Day, T. & Wild, G. Evolution of cooperation in a finite homogeneous graph. *Nature* **447**, 469–472 (2007).

[6] Nowak, M. A., Tarnita, C. E. & Antal, T. Evolutionary dynamics in structured populations. *Phil. Trans. R. Soc. B* **365**, 19–30 (2010).

[7] Antal, T., Ohtsuki, H., Wakeley, J., Taylor, P. D. & Nowak, M. A. Evolution of cooperation by phenotypic similarity. *Proc. Natl. Acad. Sci. USA* **106**, 8597–8600 (2009).

[8] Li, A., Broom, M., Du, J. & Wang, L. Evolutionary dynamics of general group interactions in structured populations. *Phys. Rev. E* **93**, 022407 (2016).

[9] Allen, B., Lippner, G., Chen, Y.-T., Fotouhi, B., Momeni, N., Yau, S.-T. & Nowak, M. A. Evolutionary dynamics on any population structure. *Nature* **544**, 227–230 (2017).

[10] Tarnita, C. E., Antal, T., Ohtsuki, H. & Nowak, M. A. Evolutionary dynamics in set structured populations. *Proc. Natl. Acad. Sci. USA* **106**, 8601–8604 (2009).

[11] Santos, M. D., Pinheiro, F. L., Santos, F. C. & Pacheco, J. M. Dynamics of n-person snowdrift games in structured populations. *J. Theor. Bio.* **315**, 81–86 (2012).

[12] Perc, M. & Szolnoki, A. Coevolutionary games - a mini review. *BioSystems* **99**, 109–125 (2010).

[13] Holme, P. & Saramäki, J. Temporal networks. *Phys. Rep.* **519**, 97–125 (2012).

[14] Pinheiro, F. L., Santos, F. C. & Pacheco, J. M. Linking individual and collective behavior in adaptive social networks. *Phys. Rev. Lett.* **116**, 128702 (2016).

[15] Szabó, G. & Fáth, G. Evolutionary games on graphs. *Phys. Rep.* **446**, 97–216 (2007).

[16] Traulsen, A., Nowak, M. A. & Pacheco, J. M. Stochastic dynamics of invasion and fixation. *Phys. Rev. E* **74**, 011909 (2006).

[17] Hauert, C. & Szabó, G. Game theory and physics. *Am. J. Phys.* **73**, 405 (2005).

[18] Szolnoki, A. & Perc, M. Emergence of multilevel selection in the prisoner's dilemma game on coevolving random networks. *New J. Phys.* **11**, 093033 (2009).

[19] Newth, D. & Cornforth, D. Asynchronous spatial evolutionary games. *Biosystems* **95**, 120–129 (2009).

[20] Santos, F. C., Pacheco, J. M. & Lenaerts, T. Evolutionary dynamics of social dilemmas in structured heterogeneous populations. *Proc. Natl. Acad. Sci. USA* **103**, 3490–3494 (2006).

[21] Rapoport, A. & Chammah, A. *Prisoner's Dilemma: A Study in Conflict and Cooperation.* Ann Arbor paperbacks (University of Michigan Press, Ann Arbor, 1965).

[22] Nowak, M. A. & May, R. M. Evolutionary games and spatial chaos. *Nature* **359**, 826–829 (1992).

[23] Santos, F. C. & Pacheco, J. M. Scale-free networks provide a unifying framework for the emergence of cooperation. *Phys. Rev. Lett.* **95**, 098104 (2005).

[24] Szabó, G. & Tőke, C. Evolutionary prisoner's dilemma game on a square lattice. *Phys. Rev. E* **58**, 69–73 (1998).

[25] Barabási, A.-L. & Albert, R. Emergence of scaling in random networks. *Science* **286**, 509–512 (1999).

[26] Erdős, P. & Rényi, A. On the evolution of random graphs. *Publ. Math. Inst. Hung. Acad. Sci.* **5**, 17–60 (1960).

[27] Watts, D. J. & Strogatz, S. H. Collective dynamics of 'small-world' networks. *Nature* **393**, 440–442 (1998).

[28] Holme, P. & Kim, B. J. Growing scale-free networks with tunable clustering. *Phys. Rev. E* **65**, 2–5 (2002).
